# TDP-43 mediated blood-brain barrier permeability and leukocyte infiltration promote neurodegeneration in a low-grade systemic inflammation mouse model

**DOI:** 10.1186/s12974-020-01952-9

**Published:** 2020-09-26

**Authors:** Frank Zamudio, Anjanet R. Loon, Shayna Smeltzer, Khawla Benyamine, Nanda K. Navalpur Shanmugam, Nicholas J. F. Stewart, Daniel C. Lee, Kevin Nash, Maj-Linda B. Selenica

**Affiliations:** 1 Byrd Alzheimer’s Institute, University of South Florida, 4001 E. Fletcher Ave, Tampa, FL 33613 USA; 2 Department of Pharmaceutical Sciences, College of Pharmacy, University of South Florida, 12901 Bruce B. Downs Blvd, Tampa, FL 33613 USA; 3 Department of Neurology, Massachusetts General Hospital Research Institute, Charlestown, MA 02129 USA; 4 Department of Molecular Pharmacology and Physiology, Morsani College of Medicine, University of South Florida, 12901 Bruce B. Downs Blvd, Tampa, FL 33612 USA; 5 Sanders-Brown Center on Aging, Department of Molecular and Cellular Biochemistry, College of Medicine, University of Kentucky, Lexington, KY USA

**Keywords:** TDP-43, Synaptic dysfunction, Microglial activation, Astrocytosis, Systemic inflammation, Neurovascular unit, Blood-brain barrier

## Abstract

**Background:**

Neuronal cytoplasmic inclusions containing TAR DNA-binding protein 43 (TDP-43) are a neuropathological feature of several neurodegenerative diseases, including amyotrophic lateral sclerosis (ALS), frontotemporal dementia (FTD), and Alzheimer’s Disease (AD). Emerging evidence also indicates that systemic inflammation may be a contributor to the pathology progression of these neurodegenerative diseases.

**Methods:**

To investigate the role of systemic inflammation in the progression of neuronal TDP-43 pathology, AAV9 particles driven by the UCHL1 promoter were delivered to the frontal cortex of wild-type aged mice via intracranial injections to overexpress TDP-43 or green fluorescent protein (GFP) in corticospinal motor neurons. Animals were then subjected to a low-dose (500 μg/kg) intraperitoneal *E. coli* lipopolysaccharide (LPS) administration challenge for 2 weeks to mimic a chronically altered low-grade systemic inflammatory state. Mice were then subjected to neurobehavioral studies, followed by biochemical and immunohistochemical analyses of the brain tissue.

**Results:**

In the present study, we report that elevated neuronal TDP-43 levels induced microglial and astrocytic activation in the cortex of injected mice followed by increased RANTES signaling. Moreover, overexpression of TDP-43 exerted abundant mouse immunoglobulin G (IgG), CD3, and CD4+ T cell infiltration as well as endothelial and pericyte activation suggesting increased blood-brain barrier permeability. The BBB permeability in TDP-43 overexpressing brains yielded the frontal cortex vulnerable to the systemic inflammatory response following LPS treatment, leading to marked neutrophil infiltration, neuronal loss, reduced synaptosome-associated protein 25 (SNAP-25) levels, and behavioral impairments in the radial arm water maze (RAWM) task.

**Conclusions:**

These results reveal a novel role for TDP-43 in BBB permeability and leukocyte recruitment, indicating complex intermolecular interactions between an altered systemic inflammatory state and pathologically prone TDP-43 protein to promote disease progression.

## Background

TAR (transactive response) DNA-binding protein 43 kDa (TDP-43), encoded by the *TARDBP* gene, is a 43-kDa nuclear protein that belongs to the heterogeneous nuclear ribonucleoproteins (hnRNPs) family that binds RNA [1]. Several studies have shown the diverse transcription regulation functions of TDP-43, as it is abundantly expressed in nearly all tissues [2]. For example, TDP-43 can regulate gene expression of proteins through mRNA stabilization such as histone deacetylase 6 (HDAC6), Tbc1d1, acrosomal protein SP10, and various synaptic proteins, thus indirectly targeting several cellular pathways that affect cell survival, mitochondrial function, metabolism, and synaptic function depending on where TDP-43 is expressed [3–6]. In 2006, TDP-43 emerged as a prime component of ubiquitinated, insoluble inclusions found in the brains of patients diagnosed with amyotrophic lateral sclerosis (ALS), a progressive motor syndrome that causes muscle weakness and atrophy, and frontotemporal dementia (FTD), a disease involving a variety of cases with behavioral and language impairment that is often accompanied by atrophy of the frontal and temporal lobes [7, 8]. In support, increased brain atrophy has been found in patients that present with both ALS and FTD [9]. Further, elevated TDP-43 levels have been reported in the CSF of FTD and ALS patients [10]. And most recently, intracellular inclusions consisting of TDP-43 have been found in 57% of Alzheimer’s disease (AD) cases studied, further highlighting the importance of pathological TDP-43 in the progression of a range of neurological diseases [11].

Notably, many patients suffering from neurological disorders present a persistently heightened systemic inflammatory state referred to as low-grade systemic inflammation which is a risk factor for morbidity and mortality in the elderly [12]. Older patients with dementia, and even old individuals without dementia, experience low-grade systemic inflammation characterized by increased high-sensitivity C-reactive protein (hs-CRP) levels associated with increased cognitive decline [13]. A study by Miller et al. showed an increased prevalence of the non-thyroid autoimmune disease in patients diagnosed as frontotemporal dementia with motor neuron disease and symptomatic *C9ORF72* mutation carriers [14]. High levels of wide-range CRP and fibrinogen, as well as increased erythrocyte sedimentation rate and neutrophil-to-lymphocyte values, were detected in ALS and its presence correlates with a negative prognosis [15]. ALS blood samples also consistently portray changes in systemic inflammatory markers (i.e., IFN-λ, IL-2, IL-8) and peripheral cell populations (lymphocytes and monocytes) [16–18]. Further linking an interaction of systemic inflammation and neurodegenerative disease, epidemiological studies support the idea of an intrinsically altered immune system in ALS and FTD patients, since diagnoses are often associated with a previous diagnosis of an autoimmune disorder [19]. Combined, these data warrant investigation on how alterations in the systemic inflammatory response, if unchecked, can influence neuroinflammation and affect disease symptoms, especially to disease-associated proteins. For example, in several mouse models of Alzheimer’s disease, *E. coli* lipopolysaccharides (LPS)-induced inflammation can exacerbate pathological accumulation of disease-associated proteins, including tau and β-amyloid [20, 21]. Moreover, inflammatory agents such as LPS and tumor necrosis factor (TNF-α) have been found to induce the cytoplasmic accumulation and aggregation of TDP-43, altering its localization and function, ultimately causing cell death in cell culture and spinal cords of transgenic TDP-43 A315T mice [22]. TDP-43 can also become extracellular through DnaJ/Hsc70 complexes and through exosomes which can either allow its clearance or exacerbate spreading [23, 24].

Although TDP-43 is linked to several diseases that present an altered systemic inflammatory response (i.e., ALS, FTD, AD), the relationship between the two has not been fully explored. Here, we investigate the effect of chronic low-grade systemic inflammation, using *E. coli* LPS, in the progression of TDP-43 proteinopathies in neurons of the frontal cortex, a brain region affected by many of these diseases. Through neurobehavioral, biochemical, and histochemical assessments, our findings suggest that elevated TDP-43 levels render the brain vulnerable to the systemic immune response during inflammation by promoting BBB permeability and impairing components of the neurovascular unit.

## Materials and methods

### Virus preparation

TDP-43 (FLAG-tagged) or GFP were cloned into the Hind III and Sac I sites of the rAAV vector pTR-MCS under the control of the ubiquitin carboxy-terminal hydrolase L1 (UCHL1) promoter. rAAV serotype 9 viruses were generated using pAAV9 and pXX6 in Hek293 cells as described previously [25].

### Animals and stereotaxic intracranial AAV injection procedure

**Fig. 1:**
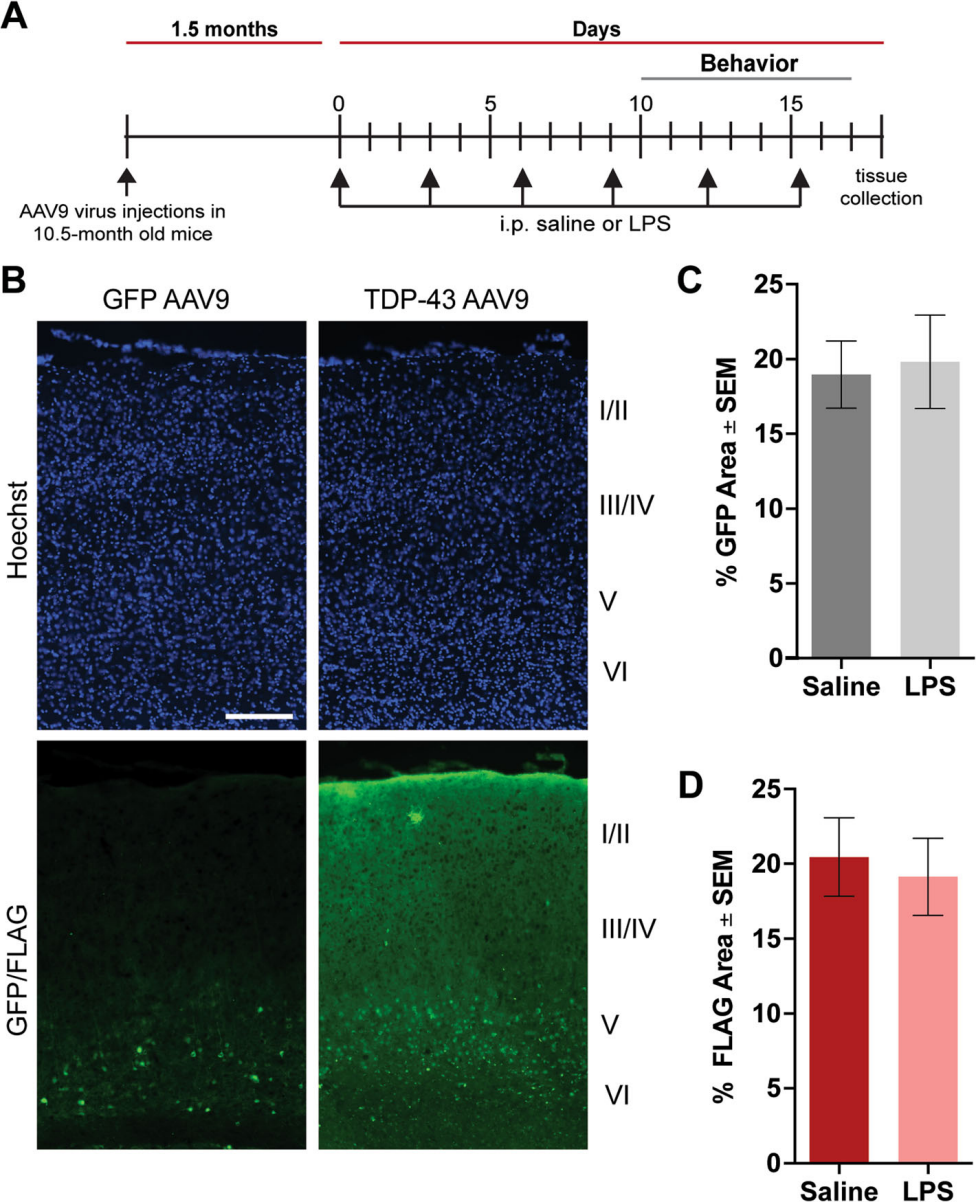
Schematic of experimental plan and analysis of viral expression in the frontal cortex of mice. **a** Schematic of experimental design involving intraperitoneal (I.P.) saline or LPS treatment in this study. **b** Representative images of the virus spreading for the study. Hoechst staining represents nuclear labeling. Scale bar = 1000 μm. **c** Percent area positively stained for GFP in the frontal cortex. **d** Percent area positively stained for FLAG in the frontal cortex

Wild-type mice (equally stratified for sex, male vs. female) were bred in the vivarium of the Byrd Alzheimer's Institute, USF Health. The injection procedure was performed using the convection-enhanced delivery method as described previously [25]. Briefly, mice were anesthetized with 1.5% isoflurane and ketamine (90–150 mg/kg)/xylazine (5–10 mg/kg) in 100% oxygen, then secured into a stereotaxic apparatus. The coordinates of injection into the cortex were as follows: anteroposterior, + 2.2 mm; lateral, ± 1.7 mm, dorsoventral, − 3.0 mm, from bregma. A microsyringe injector and controller (Stoelting, Wood Dale, IL) were used to inject 2 μL of virus (2 × 10^12^ viral particles/mL) at a constant rate of 2.5 μL/min through a CED needle in each placement. The needle was kept in place for 1 min following the injection and then was raised slowly. Either rAAV9-GFP or rAAV9-TDP-43 under the UCHL1 promoter was injected bilaterally in the cortex of wild-type mice at 10.5 months (*n* = 6 mice/group). Virus transduction through anterior cortical layer V and IV neurons was determined based on cell type and boundaries of cortical layers I–VI following *Allen Mouse Brain Atlas* (Institute 2011) (Fig. 1b). This area was targeted during image acquisition for histology analyses.

Animal procedures were performed in accordance with the recommendations of the National Research Council’s “Guide for the Care and Use of Laboratory Animals” and were previously approved by the University of South Florida Institute of Animal Care and Use Committee (IACUC).

### Mouse intraperitoneal (i.p.) injection treatment

To model low-grade systemic inflammation, we challenged the mice with a modified *E. coli* lipopolysaccharide (LPS) treatment regime as previously published [26]. Briefly, 2 weeks prior to sacrifice, mice groups were given intraperitoneal (i.p) injections of *E. coli* lipopolysaccharides (LPS; 500 μg/kg) every 3 days. This way, four treatment groups were created: GFP AAV9 (i.p. saline), GFP AAV9 (i.p. LPS), TDP-43 AAV9 (i.p. saline), and TDP-43 AAV9 (i.p. LPS) (*n* = 6 mice/group).

### Mouse behavior

Mouse behavior was performed during the last week of LPS treatment prior to sacrifice. All behavioral tests are blinded to genotype and treatment to the investigator.

#### Rotarod

Motor coordination and learning performance were assessed by placing mice onto an accelerating circular rod (Maze Engineers, Boston, MA, USA). The time until falling was recorded for each mouse. Mice were given four trials each day for 2 consecutive days. Performance in the first trial of day 1 serves as a measure of baseline locomotor coordination, whereas improvement in the latency to fall within each day (short-term learning) and between days (long-term learning) serve as measures of motor learning.

#### Grip strength

Grip strength was measured using a grip strength meter (Harvard Apparatus, 76-1066, Holliston, MA, USA). Mice were held by the base of the tail and allowed to grasp the bar of the meter with their front or back paws. The mouse was then pulled gently backward away from the bar slowly to allow the mouse to develop a resistance against the pulling force. The peak force given by the meter was recorded from their front and back paw measurements.

#### Radial arm water maze

The radial arm water maze consists of a 1-m pool with six swim paths radiating out of an open central area, with a hidden escape platform located at the end of one of the arms. On each trial, the mouse was allowed to swim in the pool for up to 60 s to find the escape platform. Incorrect arm entries or failure to select an arm for 15 s were counted as errors. For a given mouse, the platform was located in the same arm on each trial, but the start arms were varied for each trial so that mice rely upon spatial cues to solve the task. On day 1, mice were given 15 trials, each block consisting of three trials (five blocks in total) alternating between a visible platform and a hidden platform. The following day, mice were given 15 additional trials (five blocks), all using a hidden platform. The goal arm location for sequential mice was different to avoid odor cues from revealing the goal arm. On the last day of testing, all animals were tested in the open pool task with a visible platform to ascertain their vision and ability to climb on the platform. To do so, the platform was elevated above the water surface and had an attached flag. Additionally, all visual cues were removed so that mice relied only on their sight to find the platform. Latency to find and ascend the platform was recorded (60 s maximum).

### Tissue collection

Two months post intracerebral injection, mice were weighed, overdosed with a euthanizing solution containing pentobarbital, and perfused with 25 mL of 0.9% saline solution. Brains were collected following saline perfusion and were hemisected down the sagittal midline. One hemisphere was dissected and frozen on dry ice for biochemical studies. The second hemisphere was immersion fixed in 4% paraformaldehyde for 24 h and cryoprotected in successive incubations of 10%, 20%, and 30% solutions of sucrose for 24 h in each solution. Subsequently, the fixed hemispheres were frozen on a cold stage and sectioned in the horizontal plane (25 μm thickness) using a sliding microtome. Brain sections were stored in Dulbecco’s phosphate-buffered saline (DPBS) with 10 mM sodium azide solution at 4 °C for immunohistochemistry.

### Immunohistochemistry

Six sections, representative of the brain, were chosen for histochemical analyses. For bright-field microscopy, floating sections from all animals were placed in multi-sample staining trays, and endogenous peroxidase activity was blocked (10% methanol, 3% H_2_O_2_ in PBS, 15 min). Tissue samples were permeabilized (with 0.2% lysine, 1% Triton X-100 in PBS, 30 min) and incubated overnight in primary antibody for HRP-conjugated mouse IgG (Millipore, 1:1000), CD3 (AbD serotec; 1:10,000), CD4 (AbD serotec; 1:30,000), Ly6B.2 (Bio-Rad; 1:3000), or biotinylated NeuN (Millipore; 1:30,000). Sections were rinsed in PBS, then incubated in corresponding biotinylated secondary antibodies for 2 h. Sections incubated with biotinylated NeuN proceeded directly to the enzyme conjugation step. Following incubation, tissue sections were rinsed in PBS and incubated with Vectastain Elite ® ABC kit (Vector Laboratories Burlingame, CA, USA) for enzyme conjugation. Finally, sections were developed using 0.05% diaminobenzidine, 0.5% Ni^++^, and 0.03% H_2_O_2_. Tissue sections were then mounted onto slides, dehydrated, and cover-slipped. Each immunohistochemical stain omitted some sections from primary antibody incubation to evaluate the nonspecific reaction of the secondary antibody. NeuN slides were counterstained with 0.05% cresyl violet.

For fluorescence microscopy, tissue sections were permeabilized as previously described, then incubated with the following primary antibodies overnight: FLAG (1:500, Sigma), TDP-43 (1:1000, ProteinTech), Iba1 (1:500, Wako), CD11b (1:500, Abcam), MHCII (BD Biosciences, 1:500), GFAP (1:500, Dako), and CD45 (1:500, Thermo Scientific). The next day, tissue sections were washed and placed in respective Alexa Fluor secondary antibodies (1:500, Invitrogen) for 2 h. Then, tissue sections were washed, mounted, and cover-slipped using ProLong Gold anti-fade reagent (Thermo Scientific) and counterstained with Hoechst 33342 where indicated.

### Prussian blue staining

Tissue sections were mounted on slides and dried overnight. The next day, sections were rehydrated in distilled water for 30 s then incubated in a solution containing 2% concentrated HCl and 2% potassium ferrocyanide for 15 min. Thereafter, sections were rinsed two times in distilled water for 30 s followed by rinsing in tap water for 5 min. Finally, slides were dehydrated by dipping 8 times in 95% ethanol and 8 times through two changes in 100% ethanol and cleared in xylenes 3 times for 5 min followed by cover-slipping using DPX.

### Tissue imaging, quantification, and analysis

Tissue sections stained for CD4 and NeuN were imaged using the Carl Zeiss AxioImager.Z1 microscope (Oberkochen, Germany) using a × 20 objective. Tissue sections stained for TDP-43, GFAP, Iba1, CD11b, and CD45 were imaged using the Zeiss LSM 880 (Oberkochen, Germany) at × 3 objective. GFAP, Iba1, CD11b, MHCII, and CD45 levels (integrated density) in tissue sections were quantified using ImageJ analysis software with six representative regions of interest per mouse (National Institutes of Health). To determine the levels of extranuclear TDP-43, × 63 images from brain sections stained with TDP-43 and DAPI were converted to 8-bit and processed by Gaussian blur to remove background (radius = 50). Then a mask was created using the DAPI channel and subtracted from the TDP-43 channel to remove the nuclear signal. Finally, integrated density values were calculated from these images using a threshold of positive staining that was kept constant throughout the analysis. The number of neurons stained with NeuN and cresyl violet in the frontal cortex of mice was counted manually by a blind investigator in at least six 300-μm regions of interest per mouse around the site of injection. Ly6B.2, CD3, and CD4+ cells were counted manually in whole-brain sections from at least six sections per mouse used as representative.

### Western blotting

The frontal cortex and hippocampus of mice were homogenized in ice-cold RIPA buffer and sonicated for 30 s. Thereafter, the tissue homogenate was centrifuged at 38,000×*g* for 30 min. The supernatant was used for biochemical analyses. The protein concentration of each sample was measured using BCA assay. To obtain insoluble TDP-43 fraction, pellets were washed in RIPA buffer to eliminate remaining soluble proteins and centrifuged at 100,000*g* for 30 min at 4 °C. Thereafter, the supernatant was discarded and urea buffer (7 M urea, 2 M thiourea, 4% CHAPS, 30 mM Tris, pH 8.5) was added to the pellet, sonicated, and centrifuged at 100,000*g* for 30 min at room temperature. Protein concentration was determined by Bradford assay. Lysates were separated by SDS-PAGE and transferred to a PVDF membrane. Human TDP-43 (Sigma), total TDP-43 (ProteinTech), GFAP (1:1000, Dako), PSD95 (1:1000, Millipore), SNAP-25 (1:1000, Abcam), SNAP-23 (1:1000, Synaptic Systems), Syntaxin-1A (1:1000, Cell Signaling), laminin (ab11575), ICAM1 (1:1000, R & D Systems), occludin (1:1000, Invitrogen), ZO-1 (1:1000, Invitrogen), claudin 5 (1:1000), VCAM (1:1000), caveolin (1:1000, Santa Cruz), CD13 (1:1000, Abcam), PDGFRβ (Abcam), GAPDH (1:5000, Abcam), and actin (1:5000, Abcam) levels were assessed by addition of primary antibodies, species-appropriate secondary antibodies (Southern Biotech, 1:1000 or LICOR IR Dye 680/800, 1:10,000), and exposed to ECL for chemiluminescent detection or imaged using a LICOR machine.

### Multiplex chemokine/cytokine assay

The concentrations of eotaxin, IL-1α, Il-1β, IL-10, VEGF, MIP-1α, RANTES, and KC-GRO were measured using the mouse cytokine/chemokine panel in mouse frontal cortex tissue lysate (MILLIPLEX MAP kit; Millipore, Billerica, MA, USA). Briefly, the Bio-Plex Suspension Array System (Bio-Rad Laboratories) was calibrated using CAL2 with the high PMT setting of the Bio-Plex calibration kit, and standard sample preparation was performed according to the manufacturer’s directions. The filter plate was prewetted with wash buffer and vacuum-filtered before adding standard, control, or study samples to the appropriate wells. Mixed capture beads were then added to each well, and the plates were incubated overnight at 4 °C with shaking. After two washes, 25 μL of detection antibody was added to each well, incubated for 1 h at room temperature, and then treated with 25 μL of streptavidin-phycoerythrin for 30 min at room temperature. The plate was washed twice, and 150 μL of the Bio-Plex sheath fluid assay buffer was added to each well and read using the Bio-Plex Suspension Array System software (Bio-Rad Laboratories) per the kit instructions. The concentration of each analyte was calculated according to the standard curve.

### Statistical analyses

Two-tailed Student’s *t* test, one-way ANOVA or two-way repeated-measures ANOVA with Fisher’s Least Significant Difference (LSD), and Tukey or Bonferroni’s post hoc tests were used as detailed in the figure legends. Values were considered significant if *p* < 0.05. Graphs were generated using GraphPad Prism 8.0 analysis software. *N*-value in this study is depicted as the number of animals and indicated in the figure legends.

## Results

### In vivo TDP-43 overexpression promotes the formation of insoluble inclusions

To investigate the interaction between TDP-43 pathology and low-grade systemic inflammation, we used AAV-serotype 9 (AAV9) viruses to drive the overexpression of FLAG-tagged wild-type TDP-43 (TDP-43 AAV9) or GFP under the UCHL1 promoter in the frontal cortex 10.5-month-old wild-type mice for 2 months. UCHL1 is abundantly expressed in corticospinal motor neurons (CSMN) that become affected in TDP-43 proteinopathies which made its promoter ideal for our study [27–29]. Next, to model low-grade systemic inflammation, we further challenged the mice with a modified *E. coli* lipopolysaccharide (LPS) treatment regimen as described in the “Methods” section and Fig. 1a. LPS is an endotoxin frequently used to induce an immune response through toll-like receptor 4 (TLR4) binding and we surmised that a low LPS dose and spread out treatment plan would most resemble a chronic physiological inflammatory response [30]. After brain tissue collection, we confirmed the expression of our constructs by fluorescent GFP or FLAG signal combined with nuclear Hoechst staining. Further, utilization of Allen’s Brain Atlas (atlas.brain-map.org) allowed us to determine viral transduction throughout layers of the cortex (Fig. 1b). The quantification of the percent area positive for GFP or TDP-43 (FLAG) revealed that 15–20% of the frontal cortex was successfully transduced by the AAVs, mostly, throughout neuronal layers 5 and 6 (Fig. 1c, d).

**Fig. 2:**
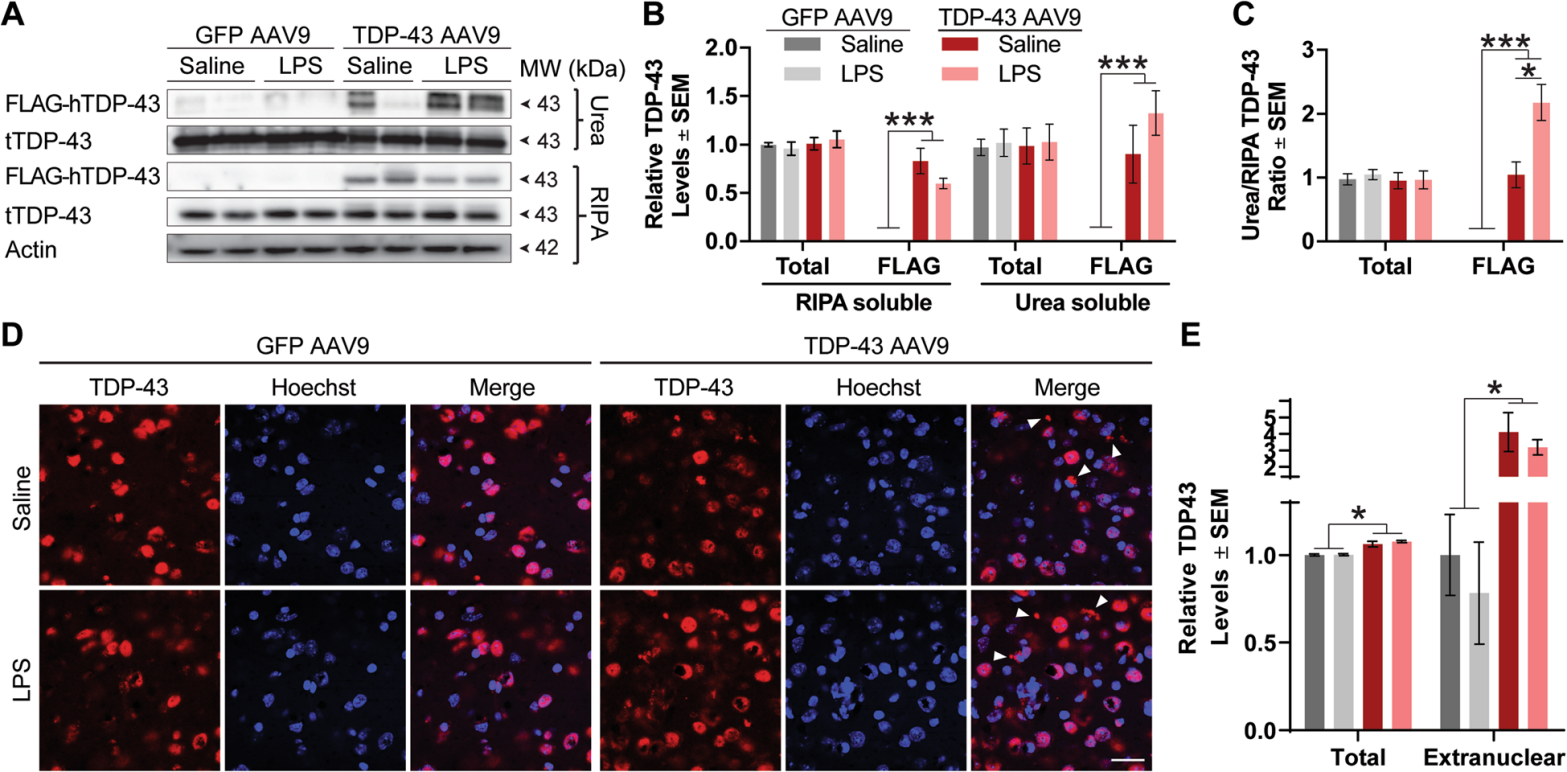
TDP-43 overexpression leads to the formation of extranuclear inclusions. **a** Western blot of total soluble and insoluble TDP-43 levels in the mouse frontal cortex. **b** Quantification of total soluble and insoluble TDP-43 levels. Statistical analysis was carried out using a one-way ANOVA with Bonferroni post hoc test. **c** Quantification of the insoluble/soluble total TDP-43 and TDP-43 ratio. **d** Representative images depicting TDP-43 localization (red channel), as observed by fluorescent immunostaining and multi-photon microscopy, in the frontal cortex of mouse brain tissue. Hoechst 33342 is used as a nuclear counterstain (blue channel). Scale bar = 20 μm. **e** Quantification of total TDP-43 and extranuclear TDP-43 normalized to GFP control. Statistical analysis was carried out using a one-way ANOVA with Bonferroni post hoc test (*n* = 4 mice/group; *p < 0.05)

To characterize the TDP-43 pathology achieved by AAV overexpression, we analyzed RIPA-soluble and urea-soluble fractions from homogenized frontal cortices. Western blotting revealed soluble and insoluble FLAG-TDP-43 levels were significantly increased, confirming the expression and formation of pathology upon TDP-43 overexpression (Fig. 2a, b). Moreover, the ratio of urea-soluble to RIPA-soluble FLAG-TDP-43 increased in the frontal cortex of LPS-treated TDP-43 AAV9 mice (Fig. 2c). Interestingly, levels of both RIPA- and urea-soluble total TDP-43 remained unaltered between groups, as also demonstrated in the ratio analysis. Although TDP-43 can control its mRNA levels through a negative feedback loop when exogenously overexpressed [31], we found that total TDP43 levels were unchanged following TDP-43 AAV9 overexpression. To further investigate this finding, we performed immunohistochemistry against total TDP-43 (red channel) and measured the signal intensity in TDP-43 AAV9 mice compared to GFP AAV mice. By doing so, we found that TDP-43 overexpression, independent of the peripheral treatment, contributed to a significant increase in total TDP-43 levels (Fig. 2d, e). Immunohistochemical analyses also revealed that overexpression of TDP-43 led to a significant increase in inclusion formation outside of the nucleus, most likely cytoplasmic (white arrows). However, low-grade systemic inflammation did not alter the inclusion burden in the frontal cortex. Further, biochemical analyses revealed no changes in TDP-43 high-molecular-weight species or C-terminal fragments following low-grade systemic inflammation (Supplementary Fig. 1a, b). Additionally, we did not detect any appreciable levels of phosphorylated TDP-43 (pS403/404 and pS409/410) in our sample (data not shown), albeit phosphorylation may not necessarily be a sole factor of TDP-43 aggregation in inclusions [32].

### Low-grade systemic inflammation impairs spatial memory in mice affected by TDP-43 overexpression

**Fig. 3:**
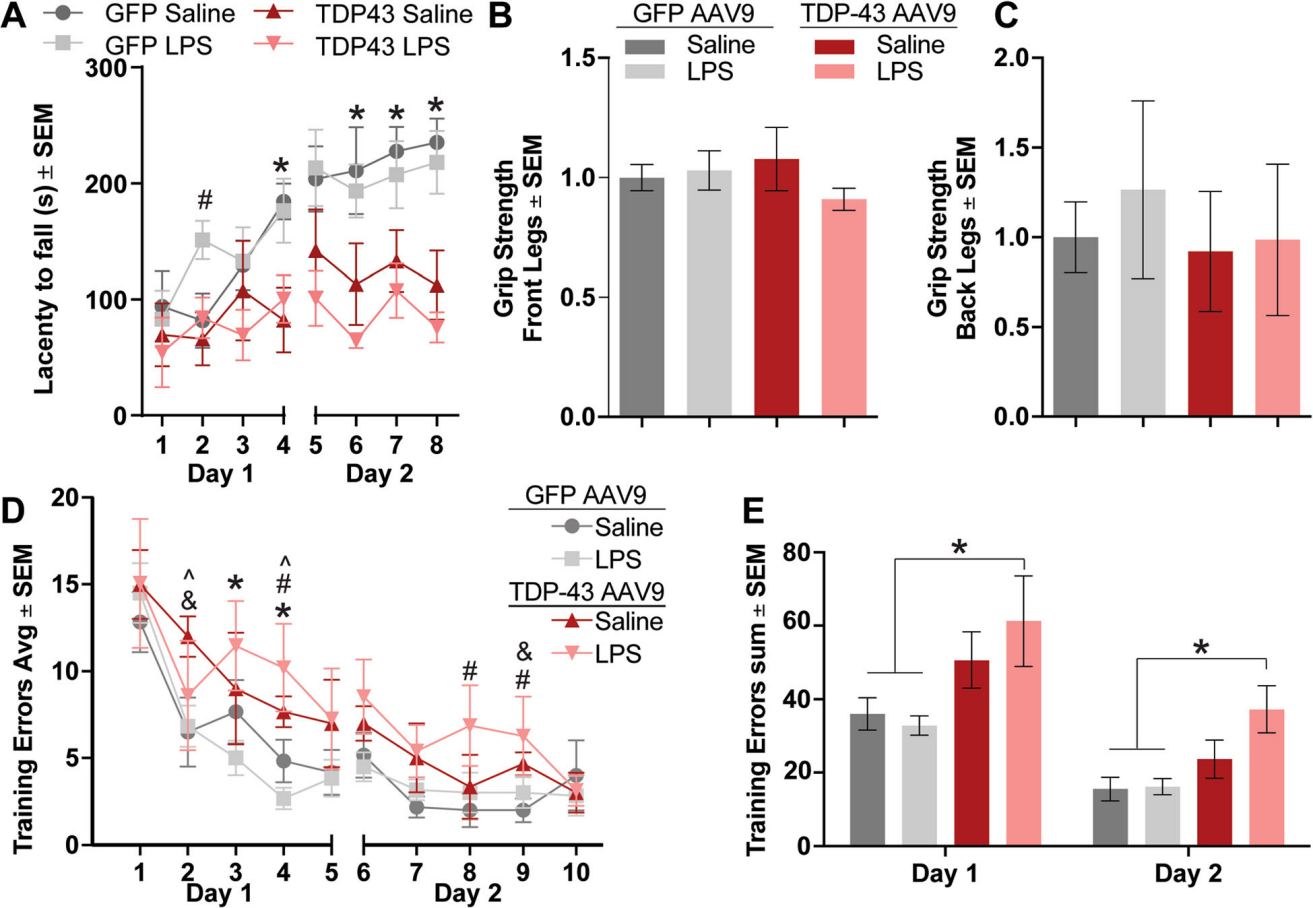
Chronic low-grade systemic inflammation impairs spatial memory in TDP-43 overexpressing mice. **a** Time spent on the rotating rod. Statistical analysis was carried out using a repeated measures two-way ANOVA with Fisher’s least significant difference (LSD) post hoc test (*n* = 6 mice/group, *p < 0.05 between GFP and TDP-43 regardless of i.p. treatment; #*p* < 0.05 between GFP-LPS against all other groups). **b** Grip strength of mice front paws performance. Statistical analysis was carried out using a one-way ANOVA with Bonferroni post hoc test (*n* = 6 mice/group). **c** Grip strength of mice hind paws performance. Statistical analysis was carried out using a one-way ANOVA with Bonferroni post hoc test (*n* = 6 mice/group). **d** Radial arm water maze performance. Statistical analysis was carried out using a repeated measures two-way ANOVA with Fisher’s least significant difference (LSD) post hoc test (*n* = 6 mice/group; *p < 0.05 between GFP-LPS and TDP-43-LPS; #*p* < 0.05 between GFP-saline and TDP-43-LPS; and *p* < 0.05 between GFP-LPS and TDP-43-saline; ^*p* < 0.05 between GFP-saline and TDP-43-saline). **e** The total number of errors in the 2-day radial arm water maze. Statistical analysis was carried out using a two-way ANOVA with Fisher’s least significant difference (LSD) post hoc test (*n* = 6 mice/group; *p < 0.05)

Various TDP-43 mouse models have reported muscle weakness, impaired motor learning, and deficits in learning and memory [33, 34]. We assessed our viral AAV9 mouse model with several behavioral tasks to ascertain their phenotype. Rotarod analysis revealed that TDP-43 overexpressing mice (regardless of LPS administration) performed similarly to GFP overexpressing littermates during the first day until the last trial, suggesting normal locomotor coordination and strength in all groups. However, mice overexpressing TDP-4 did not improve in their latency to fall from the rod on the second day, compared to control mice, indicating impaired motor learning following overexpression of TDP-43 (Fig. 3a). In support of a deficit in learning rather than coordination or strength, we also found no differences between GFP and TDP-43 AAV9 mice in grip strength (front and back legs) (Fig. 3b, c). Spatial learning and memory were assessed using the radial arm water maze, which revealed a learning impairment in TDP-43 AAV9 mice subjected to low-grade systemic inflammation (Fig. 3d, e). The LPS-treated TDP-43 AAV9 mice performed more errors in both days when compared to the LPS- (trials 3 and 4) and the saline-treated GFP AAV9 mice (trials 4, 8, and 9) (Fig. 3d). The saline-treated TDP-43 AAV9 mice also displayed a trend to more errors on day 1 of training (trials 2 and 4) but not in day 2, as compared to the saline-treated GFP AAV9 mice. No differences were observed between the saline and LPS-treated GFP AAV9 mice. Overall, LPS-treated TDP-43 AAV9 mice made significantly more errors in finding the platform in both days compared to the saline- and LPS-treated GFP AAV9 mice (Fig. 3e), indicating impaired spatial learning. However, long-term memory—assessed as the difference in the number of errors made on the last trial of day 1 and the first trial of day 2—did not statistically vary between groups, and all groups eventually achieved similar levels of learning, suggesting that the robust impairment is more likely in short-term memory processes such as those served by the prefrontal cortex. In contrast, saline-treated TDP-43 AAV9 did not make significantly more errors than the control mice. All groups showed no difference in latency of finding the visible platform in an open pool test, which indicated that the learning deficits were not attributed to visual or motor impairments (data not shown). Interestingly, LPS administration to GFP AAV9 mice did not influence spatial memory, suggesting a synergistic effect between TDP-43 pathology in the brain and low-grade systemic inflammation to affect spatial memory.

### TDP-43-induced neuronal loss is exacerbated by low-grade systemic inflammation

**Fig. 4:**
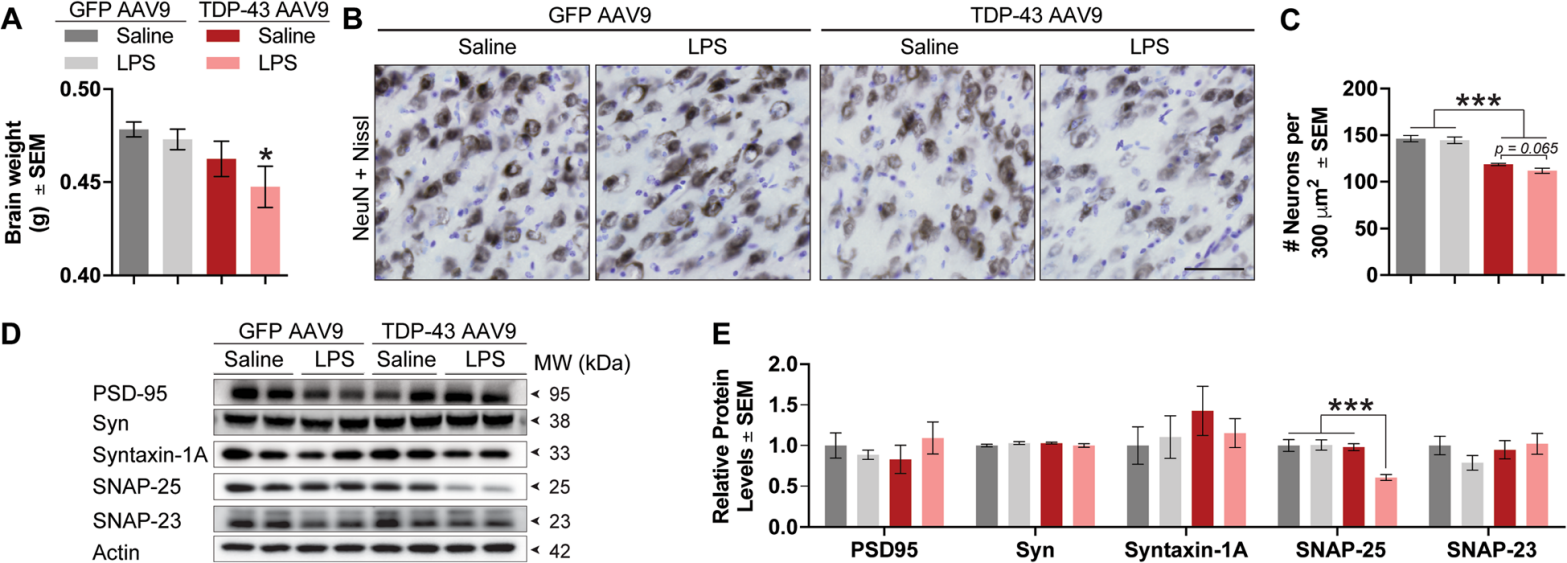
Chronic low-grade systemic inflammation promotes neuronal loss and synaptic dysfunction in TDP-43 AAV9 mice. **a** Brain weights of mice during the sacrifice. **b** Representative images depicting the number of NeuN-positive neurons. Scale bar = 20 μm. **c** Quantification of neurons per field is analyzed in the frontal cortex of the mouse brain. Statistical analysis was carried out by one-way ANOVA with Bonferroni post hoc test (*n* = 4 mice/group; *p < 0.05). **d** Western blot analysis of synaptic markers in the frontal cortex of the mouse brain. **e** Quantification of synaptic markers. Statistical analysis was carried out by one-way ANOVA with Bonferroni post hoc test (*n* = 4 mice/group; *p < 0.05)

Cognitive dysfunction is associated with neuronal loss and synaptic dysfunction [35, 36]. At sacrifice, the brains of LPS-treated TDP-43 AAV9 mice had significantly lower brain weights compared to controls, suggesting increased neurodegeneration (Fig. 4a). To determine the impact of TDP-43 overexpression and/or low-grade systemic inflammation on neuronal loss, we stained brain tissue sections with the neuronal marker NeuN, counterstained with cresyl violet, and performed stereological analyses. TDP-43 overexpression alone reduced the number of neurons in the frontal cortex of the mice and this effect was exacerbated by low-grade systemic inflammation (Fig. 4b, c). However, low-grade systemic inflammation in GFP AAV9 brains did not influence brain weight or neuronal count, suggesting that TDP-43 altered the susceptibility of the brain to the systemic inflammatory response. Next, since both TDP-43 [37–39] and LPS-mediated [40–42] neuroinflammation can alter the expression of synaptic markers that can lead to cognitive decline, we performed western blotting of several synaptic markers as a measure of synaptic dysfunction in frontal cortex lysates. While PSD95, synaptophysin, syntaxin 1-A, and SNAP-23 protein levels remain unaltered by TDP-43 overexpression alone or low-grade systemic inflammation, SNAP-25 expression levels were reduced by half in the brains of LPS-treated TDP-43 AAV9 mice (Fig. 4d, e). SNAP-25 is an essential component of N-ethylmaleimide-sensitive factor attachment protein receptor (SNARE) complexes, which mediate synaptic communication through initiating fusion of synaptic vesicles [43].

### TDP-43 overexpression induces astrogliosis and microgliosis

**Fig. 5:**
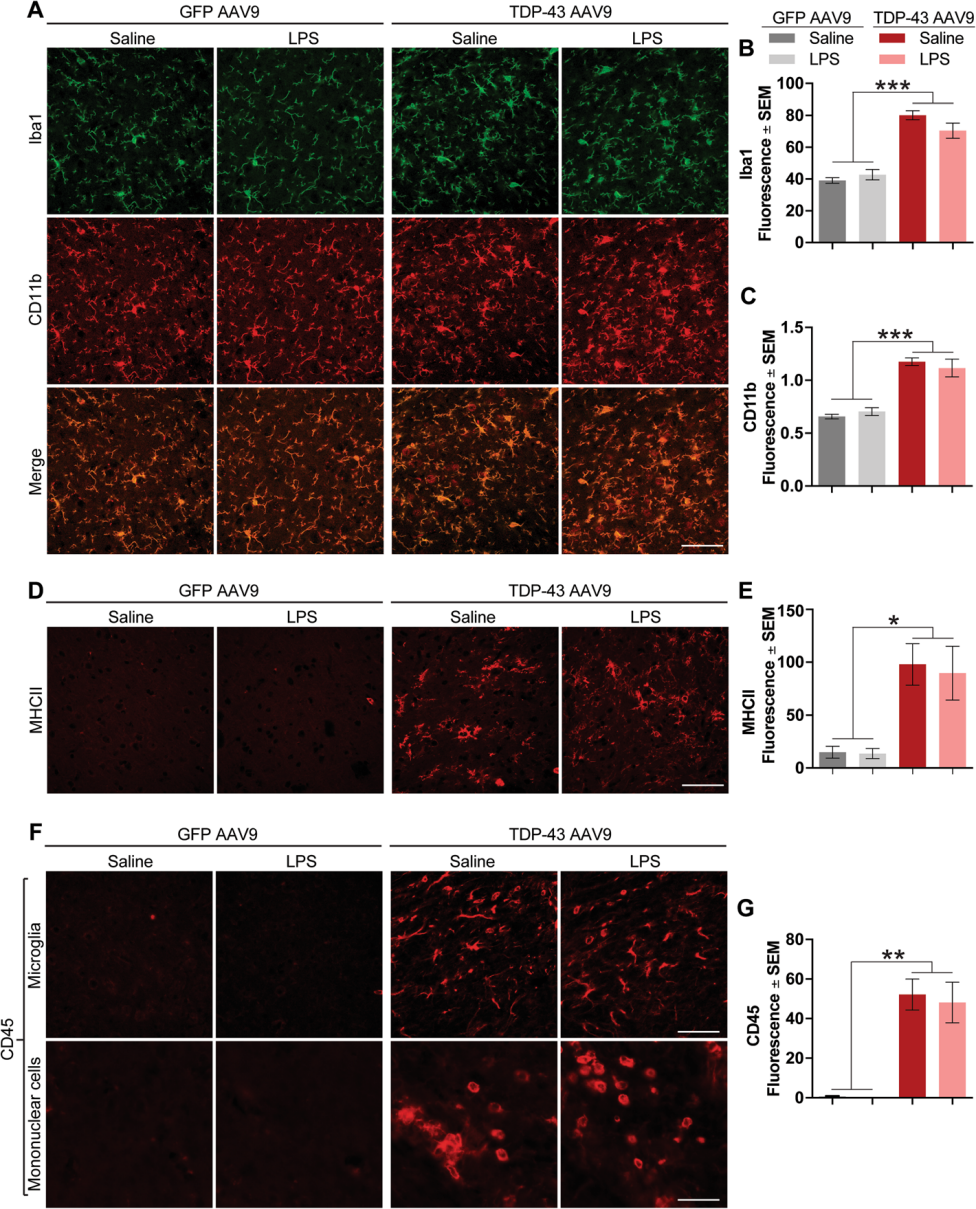
TDP-43 overexpression increases microglial activation. **a** Double immunofluorescence depicting Iba1 (green pseudocolor) and CD11b (red channel) in the frontal cortex of mouse brain tissue. Scale bar = 20 μm. **b** Quantification of Iba1 levels. Statistical analysis was carried out using a one-way ANOVA with Bonferroni post hoc test (*n* = 4 mice/group; **p < 0.01, ***p < 0.001). **c** Quantification of CD11b levels. Statistical analysis was carried out using a one-way ANOVA with Bonferroni post hoc test (*n* = 4 mice/group; **p < 0.01, ***p < 0.001). **d** Representative images depicting MHCII levels (red channel) in the frontal cortex of mouse brain tissue. Scale bar = 20 μm. **e** Quantification of MHCII levels. Statistical analysis was carried out using a one-way ANOVA with Tukey post hoc test (*n* = 4 mice/group; *p < 0.01). **f** Representative images depicting CD45 expression in microglia and mononuclear cells in the frontal cortex of mouse tissue. Scale bar = 20 μm (microglia), 10 μm (mononuclear cells). **g** CD45 fluorescence levels in the mouse frontal cortex. Statistical analysis was carried out using a one-way ANOVA with Bonferroni post hoc test (*n* = 4 mice/group; ***p < 0.001). Statistical analysis was carried out using one-way ANOVA with Bonferroni post hoc test (*n* = 4 mice/group)

Thus far, our data suggested that low-grade systemic inflammation can promote TDP-43-induced neurodegeneration, resulting in cognitive impairment. Given that the deregulation of TDP-43 can induce neuroinflammation and increase neuronal death through NF-κB signaling [28, 44], we decided to explore whether the observed effects were due to alterations in the NF-κB pathway. Our biochemical analysis demonstrated no changes in phosphorylation of NF-κB p65 or IKβα degradation within the confines of our study design (data not shown). Next, we explored the effects of systemic inflammation and/or TDP-43 overexpression in the brain in other aspects of neuroinflammation, including astrocytosis and microgliosis. Indeed, TDP-43 overexpression alone—and independent of LPS—increased astrocytic activation, as measured by increased GFAP staining intensity (Supplementary Figure 2A-B) and western blotting (Supplementary Figure 2C-D). However, neither TDP-43 overexpression nor LPS treatment altered aquaporin 4 levels, a marker for astrocytic end-feet (Supplementary Figure 2E). TDP-43 overexpression also led to marked increases in microgliosis, measured by several markers including Iba1, CD11b (Fig. 5a–c), MHCII (Fig. 5d, e), and CD45 (Fig. 5f, g). While both MHCII and CD45 markers can be expressed by microglia [45, 46], MHCII is highly expressed in dendritic cells and lymphocytes while CD45 is expressed in almost all hematopoietic cells [47–49]. Interestingly, staining for MHCII and CD45 was absent from GFP AAV9 brains and dramatically increased in TDP-43 AAV9 brains. Additionally, CD45 staining revealed that although activated microglial cells were present, other cells void of processes were highly abundant, indicating the possibility of peripheral cell infiltration. Surprisingly, low-grade systemic inflammation alone did not significantly alter astrocytosis or microgliosis. However, LPS-mediated neuroinflammation can vary dependent on the brain region, since we observed significant increases in inflammation-responsive proteins iNOS, RGS14, and phosphorylated CamKIIβ in hippocampal, but not frontal cortex, homogenates from mice in our study (Supplemental Figure 3A-B).

### TDP-43 overexpression promotes blood-brain barrier permeability

**Fig. 6:**
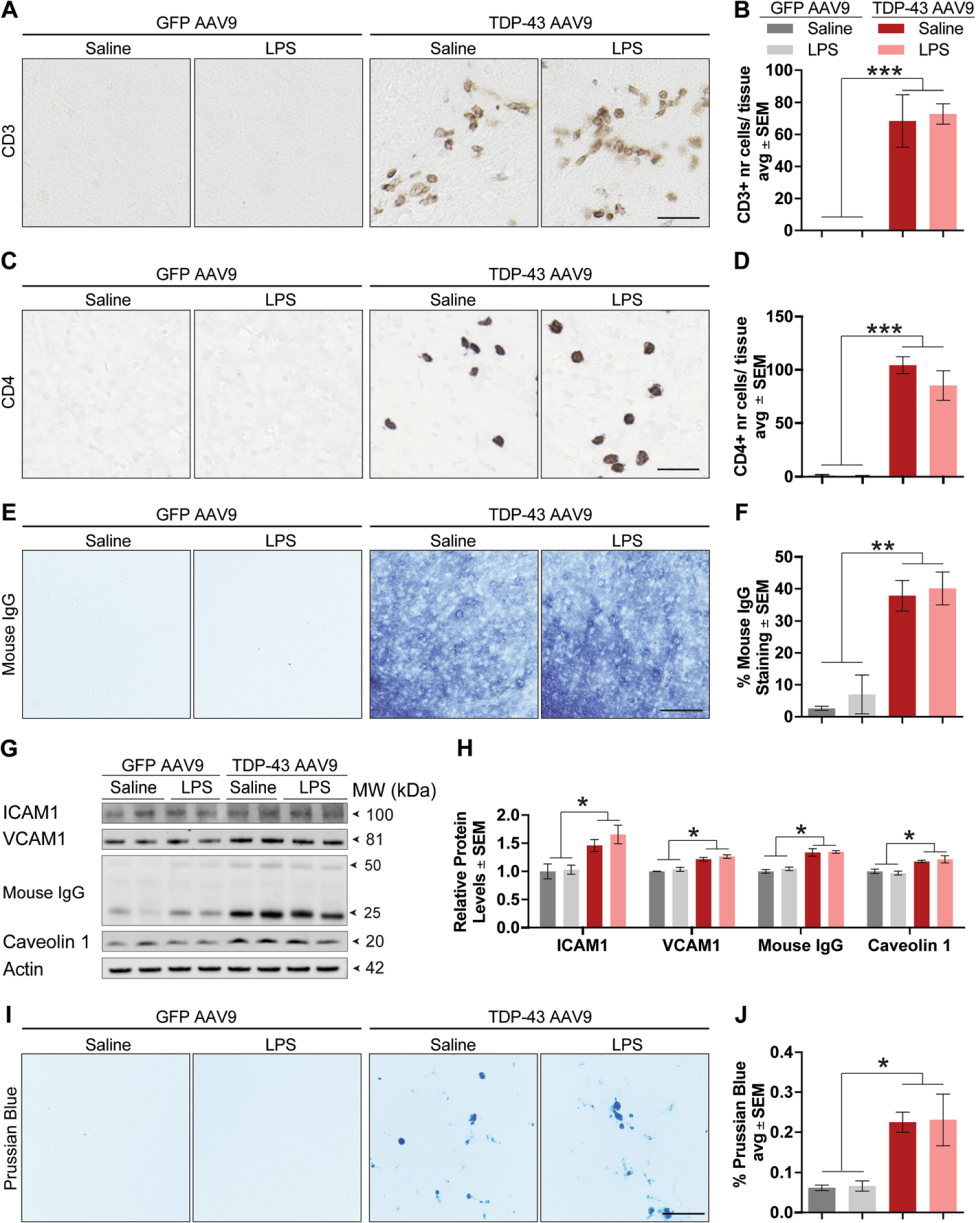
TDP-43 overexpression induces blood-brain barrier permeability. **a** Representative images depicting CD3+ T cells in the frontal cortex of mice. Scale bar = 10 μm. **b** Manual count of CD3+ T cells. Statistical analysis was carried out using one-way ANOVA with Bonferroni post hoc test (*n* = 4 mice/group, ***p < 0.001). **c** Representative images depicting CD4+ T cells in the frontal cortex of mice. Scale bar = 10 μm. **d** Manual count of CD4+ T cells. Statistical analysis was carried out using one-way ANOVA with Bonferroni post hoc test (*n* = 4 mice/group; ***p < 0.001). **e** Representative images depicting mouse IgG in the frontal cortex of mouse tissue. Scale bar = 20 μm. **f** Quantification of percent area of the frontal cortex covered by mouse IgG staining. Statistical analysis was carried out using a one-way ANOVA with Bonferroni post hoc test (*n* = 4 mice/group, ***p < 0.001). **g** Representative western blots of frontal cortex lysate probed for mouse IgG, ICAM1, VCAM1, caveolin 1, and actin. **h** Quantification of mouse IgG, ICAM1, VCAM, and caveolin 1 levels normalized to actin. Statistical analysis was carried out using a one-way ANOVA with Bonferroni post hoc test (*n* = 4 mice/group, *p < 0.05). **i** Representative images of Prussian blue staining depicting microhemorrhages in the frontal cortex of mice. Scale bar = 20 μm. **j** Quantification of percent area of the frontal cortex covered by Prussian blue-stained deposits. Statistical analysis was carried out using a one-way ANOVA with Bonferroni post hoc test (*n* = 4 mice/group, *p < 0.05)

Given the presence of mononuclear CD45-positive cells, we surmised that TDP-43 overexpression could induce peripheral cell infiltration. Therefore, brain tissue sections were labeled with CD3 and CD4 receptor antigens, both found in T lymphocytes. As suspected, TDP-43 overexpression, regardless of systemic inflammation, led to marked increases in CD3+ (Fig. 6a, b) and CD4+ (Fig. 6c, d) T lymphocyte infiltration, suggesting increased blood-brain barrier (BBB) permeability. The loss of BBB integrity was further confirmed by intense immunoglobulin G (IgG) staining along the frontal cortex of TDP-43 AAV9 mice, since the BBB restricts access of large molecules into the brain, let alone whole cells (Fig. 6e, f). IgG infiltration was also confirmed by western blotting using the same antibody. These effects were associated with increased endothelial cell activation markers, including ICAM1, VCAM, and caveolin 1 (Fig. 6,h). Moreover, TDP-43 overexpression also led to the presence of cerebral microbleeds, determined by Prussian blue staining (Fig. 6i, j). Pericyte activation, as measured by PDGFRβ levels, was also observed (Supplementary Figure 4A-B). However, tight junction proteins (ZO-1, occludin, claudin 5, and claudin 3), surprisingly, were not altered (Supplementary Figure 4C-D).

### Low-grade systemic inflammation in TDP43 overexpressing mice promotes neutrophil infiltration

**Fig. 7:**
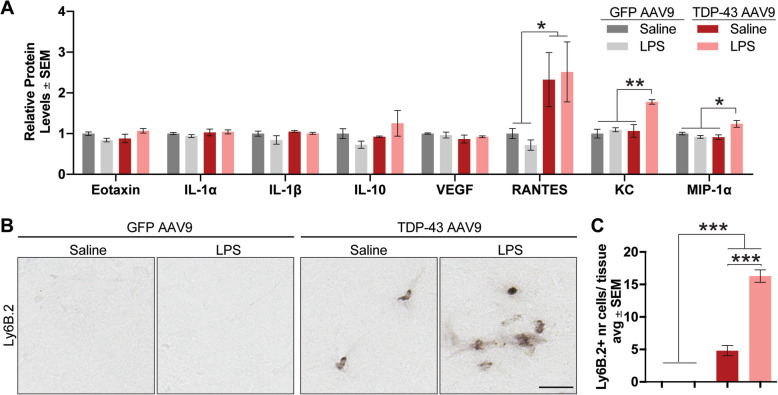
LPS-induced altered cytokine signaling during TDP-43 overexpression leads to neutrophil infiltration in the brain. **a** The concentration of eotaxin, IL-1α, IL-1β, IL-10, VEGF, RANTES, KC-GRO, and MIP-1α in frontal cortex tissue lysates. Statistical analysis was carried out using a one-way ANOVA with Bonferroni post hoc test or two-tailed *t*-tests for RANTES (*n* = 6 mice/group; *p < 0.05, ***p < 0.001). **b** Representative images of Ly6B.2+ neutrophil labeling in the frontal cortex of mice. Scale bar = 10 μm. **c** Manual count of Ly6B.2+ neutrophils. Statistical analysis was carried out using a one-way ANOVA with Bonferroni post hoc test (*n* = 4 mice/group, ***p < 0.001)

Next, we measured the levels of a range of inflammatory cytokines in frontal cortex homogenates, similar to our recent report [50]. Low-grade systemic inflammation in GFP AAV9 mice did not alter the levels of any of the cytokines measured (Fig. 7a). TDP-43 overexpression, however, increased levels of RANTES in the frontal cortex. Interestingly, the expression of KC-GRO and MIP-1α was increased in the frontal cortex of LPS-treated TDP-43 AAV9 mice only. As KC-GRO and MIP-1α are chemotactic inflammatory signals involved in neutrophil recruitment [51], we stained brain tissue sections for the Ly6B.2 receptor, which is highly expressed by murine neutrophils [52]. Immunohistochemical labeling revealed that TDP-43 overexpression alone promoted neutrophil infiltration in the brain parenchyma compared to both the saline- and LPS-treated GFP AAV9 mice (Fig. 7b, c). Meanwhile, low-grade systemic inflammation in TDP-43 AAV9 mice further exacerbated Ly6B.2+ neutrophil infiltration. The data ultimately suggests that TDP-43-induced BBB permeability may be responsible for the compounded effects of systemic inflammation on behavior impairments, neuronal loss, and synaptic dysfunction observed in LPS-treated TDP-43 AAV9 mice.

## Discussion

The etiology of several neurodegenerative diseases, including ALS, FLTD, and AD, remains elusive. Nevertheless, research has identified several mutations and proteins in the brain that accelerate disease progression and have become key pathological features, such as TDP-43. Additionally, further studies have consistently observed systemic inflammatory changes in these diseases, giving rise to the idea of, not only systemic inflammation as a trigger for neurodegeneration, but also an intrinsically altered immune system [19]. With this in mind, our study sought to investigate the interaction between the systemic inflammatory response and TDP-43-associated pathology. While several TDP-43 transgenic models are available [33, 34, 53], the global TDP-43 expression in forebrain neurons confounds the effects it may have on certain neuronal subtypes and brain regions. Therefore, an AAV9 overexpression approach proved advantageous to explore TDP-43 in a way more relevant to human disease. Altogether, we demonstrate that low-grade systemic inflammation accelerated neurodegeneration and induced cognitive impairments in TDP-43 overexpressing mice. In our study, several cell types were activated as a result of TDP-43 overexpression. Interestingly, LPS-treated TDP-43 AAV9 mice presented with an altered phenotype despite no appreciable changes in astrocytic/microglial activation, endothelial/pericytic activation, T cell infiltration, BBB leakage, or hemosiderin deposits. Though we did observe LPS-driven cytokine changes in TDP-43 overexpressing brains, suggesting inflammation-associated cascades are taking place (KC-GRO and MIP-1α). We surmise that TDP-43 alone is sufficient in mediating inflammation and probably masked the subtle effects of low-grade systemic inflammation in the frontal cortex. This is conceivable since TDP43 alone leads to significant neuronal loss.

The radial arm water maze (RAWM) is generally known to test spatial learning, which is strongly tied to hippocampal function; however, we observed significant learning impairment in the cortical TDP-43 overexpressing mice subjected to LPS. In support of our findings, studies indicate that spatial memory is not strictly tied to the hippocampus as it is not affected following hippocampal insults in certain tasks [54–58], contending that task-dependent memory demands dictate the use of distinct brain regions [59]. We reason that cortical TDP-43 in synergy with systemic inflammation directly impaired RAWM performance in mice by affecting the cortico-hippocampal networks [60–62]. In agreement, several studies demonstrate that manipulations of the cortex impaired the performance in spatial memory tasks [63–68]. Finally, we cannot exclude the role that LPS-induced hippocampal neuroinflammation observed in TDP-43 overexpressing mice has on RAWM performance. It is plausible that the altered hippocampal signaling is partially responsible for the slowed performance. Overall, our results correlate with a recent consensus study on patients with limbic age-related TDP-43 encephalopathy (LATE) demonstrating that TDP-43 neuropathology precipitated learning and spatial memory decline similar to AD in patients [69].

TDP-43 overexpression increased IgG, as well as CD3+ and CD4+ T cell infiltration, which were associated with increased endothelial signaling since both ICAM1 and caveolin 1 facilitate leukocyte infiltration in the brain [70, 71]. Further, increased VCAM1 levels were observed following TDP-43 overexpression, indicating impaired endothelial barrier integrity [72]. Therefore, it is also plausible that the TDP-43-dependent cell infiltration is partially due to the blood-brain barrier (BBB) permeability and/or dysfunction. For example, in an animal model of tau overexpression, aged mice showed increased Evans blue extravasation while also displaying significant T cell infiltration without reported tight junction loss [73]. Additionally, free hemoglobin released from extravasated red blood cells can be toxic to neurons and we observed an increase in hemosiderin deposits in areas of TDP-43 overexpression, which indicates BBB disruption. Surprisingly, we did not observe significant reductions in tight junction proteins, which could reflect limitations of the technique used; for instance, sensitivity, given that we achieved ~ 20% transduction efficiency in the frontal cortex. Regardless, our data reveal for the first time the interplay between peripheral cell infiltration signaling—as well as BBB permeability or dysfunction—and TDP-43 pathology. Since the microvasculature of the BBB allows for tight regulation of components between the blood and the brain [74, 75], rendering LPS to act mainly through endothelial receptor signaling [76], increased BBB permeability is most likely responsible for the compounded effects of LPS in TDP-43 overexpressing mice. This could be due to the infiltration of LPS or other LPS-induced blood elements [77] into the brain parenchyma.

The extravasation of T cells could exacerbate TDP-43 induced neurodegeneration. This is in agreement with studies showing that CD3+ T cell infiltration in the hippocampus promotes neuroinflammation and cognitive dysfunction in tau overexpressing models [78]. Interestingly, we also observed the presence of CD45- and MHCII-positive mononuclear cells following TDP-43 overexpression, but not GFP, indicating the possibility for infiltrating monocytes/macrophages. Several studies have linked macrophage infiltration to be detrimental in mice, for example, infiltration of macrophages in the brain after seizures in mice contributes to neuroinflammation [79]. Also, infiltrating macrophages impairs spatial memory and hippocampal long-term potentiation (LTP) in leptin receptor-deficient mice through the induction of pro-inflammatory cytokines [80]. Thus, it is plausible that infiltrating monocytes/macrophages, in addition to T cells, can drive neuroinflammation and contribute to TDP-43-mediated neurodegeneration.

Although TDP-43 overexpression alone led to peripheral cell infiltration, low-grade systemic inflammation in TDP-43 AAV9 mice increased the infiltration of neutrophils into the brain through KC-GRO and MIP-1α signaling [51, 81]. Neutrophil infiltration has been extensively associated with detrimental effects in neurodegenerative diseases [82, 83], for example, preventing neutrophil extravasation into the brain of transgenic models of amyloid deposition reduce AD neuropathology and improved cognitive function [84]. Similarly, the increased neutrophil infiltration in the TDP-43 overexpressing brain following low-grade systemic inflammation could be partly responsible for the alterations in neuronal loss and cognitive performance in our study. However, further studies are needed to understand not only the role of differential infiltrating cell populations in TDP-43-mediated BBB dysfunction but also the distinctive function of peripheral cells in the context of an altered immune response and the diseased brain.

Wild-type TDP-43 neurotoxic effects have been extensively studied in various models of overexpression [33, 34, 53], linking TDP-43-dependent neurodegeneration to transcription factor nuclear factor-κB (NF-κB) signaling [28, 85], synaptic plasticity [86], and the heat shock response [32, 87, 88]. Since ALS [89, 90], FTD [91], and AD [92] present with BBB alterations and are associated with pathological TDP-43, our data provides an important link between the two. We predict, based on our findings, that patients with brain-related TDP-43 proteinopathies have increased susceptibility to neurodegeneration, not only because of the toxicity of high TDP-43 levels but because of its global impact on brain function, rendering the immune-privileged organ vulnerable to blood components toxic to brain cells and the immune response. After all, a vast array of toxic or allergenic substances, in addition to microbes, can threaten body homeostasis and launch the immune response as a protective mechanism, which can inadvertently harm the brain. In fact, given that a number of patients with neurodegenerative diseases present with low-grade systemic inflammation [12–18], it is not surprising that the altered immune response may affect brains with BBB abnormalities and/or impair neurovascular unit function leading to increased BBB permeability and peripheral cell infiltration.

## Conclusion

Overall, our results uncover a novel role of TDP-43 in blood-brain barrier permeability and leukocyte recruitment which can compromise the brains of patients with TDP-43 proteinopathies and alter disease progression, especially in association with other inflammatory conditions. The data also shed light on TDP-43 as a factor explaining the BBB abnormalities observed in patients diagnosed with neurodegenerative diseases while also supporting a new body of research regarding the role of TDP-43 in alterations of the neurovascular unit.

## Supplementary information

**Additional file 1: Figure S1**. TDP-43 oligomers and c-terminal fragments are not altered by TDP-43 overexpression. (A) Western blots of RIPA and urea-soluble brain homogenates probed for total TDP-43 at a high exposure to visualize oligomers and C-terminal fragments. (B) Quantification of oligomers and C-terminal fragments in the RIPA and urea-soluble fractions (n = 4 mice/group). Statistical analysis was carried out using a one-way ANOVA with Bonferroni post-hoc test.

**Additional file 2: Figure S2**. TDP-43 overexpression increases astrocytic activation. (A) Representative images depicting GFAP levels (red channel) in the frontal cortex of mouse brain tissue. Scale bar = 20 μm. (B) Quantification of GFAP levels. Statistical analysis was carried out using a one-way ANOVA with Bonferroni post-hoc test (n = 4 mice/group; * p < 0.05). (C) Representative blots probed for GFAP, aquaporin 4, and actin. (D) Quantification of GFAP and (E) aquaporin 4 levels normalized to actin. Statistical analysis was carried out using a one-way ANOVA with Bonferroni post-hoc test (n = 4 mice/group; ** p < 0.01).

**Additional file 3: Figure S3**. Regional differences in LPS-driven neuroinflammation. (A) Representative blots probed for p-CamKIIβ, RGS14, and iNOS in hippocampal and frontal cortex tissue lysates. (B) Quantification of p-CamKIIβ, RGS14, and iNOS normalized to actin. Statistical analysis was carried out using one-way ANOVAs with Bonferroni post-hoc test (n = 4 mice/group).

**Additional file 4: Figure S4**. TDP-43 overexpression induces pericyte activation and does not alter tight junction protein levels. (A) Representative blots probed for CD13, PDGFRβ, and GAPDH. (B) Quantification of CD13 and PDGFRβ levels normalized to GAPDH. Statistical analysis was carried out using a one-way ANOVA with Bonferroni post-hoc test (n = 4 mice/group, * p < 0.05). (C) Representative blots probed for laminin B, ZO-1, occludin, claudin 5, claudin 3, and actin. (D) Quantification of laminin B, ZO-1, occludin, claudin 5, and claudin 3 levels normalized to actin. Statistical analysis was carried out using a one-way ANOVA with Bonferroni post-hoc test (n = 4 mice/group)

## Data Availability

All relevant data are available upon request directed to the corresponding author.

## Abbreviations

TDP-43: TAR DNA-binding protein 43
ALS: Amyotrophic lateral sclerosis
FTD: Frontotemporal dementia
AD: Alzheimer’s disease
AAV9: Adeno-associated virus 9
UCHL1: Ubiquitin carboxyl-terminal esterase L1
GFP: Green fluorescent protein
LPS: Lipopolysaccharides
RANTES: Regulated upon activation, normal T cell expressed and presumably secreted
BBB: Blood-brain barrier
IgG: Immunoglobulin G
CD3: Cluster of differentiation 3
CD4: Cluster of differentiation 4
Ly6B.2: Lymphocyte antigen 6B.2
SNAP-25: Synaptosome associated protein 25
RAWM: Radial arm water maze
hnRNPs: heterogeneous nuclear ribonucleoproteins
HDAC6: histone deacetylase 6
Tbc1d1: Tre-2/USP6, BUB2, cdc16 domain family member 1
Hs-CRP: High-sensitivity C-reactive protein
C9ORF72: Chromosome 9 open reading frame 72
IFN-λ: Interferon-gamma
IL-2: Interleukin 2
IL-8: Interleukin 8
TNF-α: Tumor necrosis factor-alpha
Hsc70: Heat shock cognate 71 kDa protein
CED: Convection-enhanced delivery
NeuN: Neuronal nuclei
Ni^++^: Nickel sulfate
H_2_O_2_: Hydrogen peroxide
MHCII: Major histocompatibility complex class II
GFAP: Glial fibrillary acidic protein
CD45: Cluster of differentiation 45
HCl: Hydrochloric acid
CD11b: Cluster of differentiation molecule 11b
BCA: Bicinchoninic assay
RIPA: Radioimmunoprecipitation assay buffer
CHAPS: 3-[(3-Cholamidopropyl) dimethylammonio]-1-propanesulfonate hydrate
SDS-PAGE: Sodium dodecyl-sulfate-polyacrylamide gel electrophoresis
PVDF: Polyvinylidene fluoride
PSD95: Post-synaptic density protein 95
SNAP-23: Synaptosome associated protein 23
ICAM1: Intercellular adhesion molecule 1
ZO-1: Zonula occludens 1
VCAM: Vascular cell adhesion molecule 1
PDGFRβ: Platelet-derived growth factor receptor beta
GAPDH: Glyceraldehyde 3-phosphate dehydrogenase
IR: Infra-red
IL-1α: Interleukin 1 alpha
Il-1β: Interleukin 1 beta
IL-10: Interleukin 10
VEGF: Vascular endothelial growth factor
MIP-1α: Macrophage inflammatory protein 1-alpha
KC-GRO: Chemokine (C-X-C motif) ligand 1
TLR4: Toll-like receptor 4
NF-κB: Nuclear factor kappa-light-chain-enhancer of activated B cells
SNARE: Synaptosome-associated protein receptor

## References

[1] Krecic A, Swanson M. hnRNP complexes: composition, structure, and function. Curr Opin Cell Biol. 1999;11(3). 10.1016/S0955-0674(99)80051-9 10395553

[2] Ayala YM, Pantano S, D’Ambrogio A (2005). Human, Drosophila, and C. elegans TDP43: nucleic acid binding properties and splicing regulatory function. J Mol Biol.

[3] Kim SH, Shanware NP, Bowler MJ, Tibbetts RS (2010). Amyotrophic lateral sclerosis-associated proteins TDP-43 and FUS/TLS function in a common biochemical complex to co-regulate HDAC6 mRNA. J Biol Chem.

[4] Chiang PM, Ling J, Jeong YH, Price DL, Aja SM, Wong PC (2010). Deletion of TDP-43 down-regulates Tbc1d1, a gene linked to obesity, and alters body fat metabolism. Proc Natl Acad Sci U S A.

[5] Abhyankar MM, Urekar C, Reddi PP (2007). A novel CpG-free vertebrate insulator silences the testis-specific SP-10 gene in somatic tissues: Role for TDP-43 in insulator function. J Biol Chem.

[6] Polymenidou M, Lagier-Tourenne C, Hutt KR (2011). Long pre-mRNA depletion and RNA missplicing contribute to neuronal vulnerability from loss of TDP-43. Nat Neurosci.

[7] Arai T, Hasegawa M, Akiyama H (2006). TDP-43 is a component of ubiquitin-positive tau-negative inclusions in frontotemporal lobar degeneration and amyotrophic lateral sclerosis. Biochem Biophys Res Commun.

[8] Neumann M, Sampathu DM, Kwong LK, et al. Ubiquitinated TDP-43 in frontotemporal lobar degeneration and amyotrophic lateral sclerosis. *Science (80- )*. Published online 2006. doi:10.1126/science.1134108. 10.1126/science.1134108 17023659

[9] Rajagopalan V, Pioro EP (2014). Distinct patterns of cortical atrophy in ALS patients with or without dementia: An MRI VBM study. Amyotroph Lateral Scler Front Degener.

[10] Steinacker P, Hendrich C, Sperfeld AD (2008). TDP-43 in cerebrospinal fluid of patients with frontotemporal lobar degeneration and amyotrophic lateral sclerosis. Arch Neurol.

[11] McAleese KE, Walker L, Erskine D, Thomas AJ, McKeith IG, Attems J (2017). TDP-43 pathology in Alzheimer’s disease, dementia with Lewy bodies and ageing. Brain Pathol.

[12] Franceschi C, Campisi J (2014). Chronic inflammation (Inflammaging) and its potential contribution to age-associated diseases. Journals Gerontol - Ser A Biol Sci Med Sci.

[13] Cervellati C, Trentini A, Bosi C (2018). Low-grade systemic inflammation is associated with functional disability in elderly people affected by dementia. GeroScience..

[14] Miller ZA, Sturm VE, Camsari GB, et al. Increased prevalence of autoimmune disease within C9 and FTD/MND cohorts Completing the picture. *Neurol Neuroimmunol NeuroInflammation*. 2016;3(6). doi:10.1212/NXI.0000000000000301. 10.1212/NXI.0000000000000301 PMC5087253 27844039

[15] Keizman D, Rogowski O, Berliner S (2009). Low-grade systemic inflammation in patients with amyotrophic lateral sclerosis. Acta Neurol Scand.

[16] Hu Y, Cao C, Qin XY, et al. Increased peripheral blood inflammatory cytokine levels in amyotrophic lateral sclerosis: A meta-analysis study. *Sci Rep*. 2017;7(1). doi:10.1038/s41598-017-09097-1. 10.1038/s41598-017-09097-1 PMC5567306 28831083

[17] Murdock BJ, Zhou T, Kashlan SR, Little RJ, Goutman SA, Feldman EL (2017). Correlation of peripheral immunity with rapid amyotrophic lateral sclerosis progression. JAMA Neurol.

[18] Zhang R, Gascon R, Miller RG (2005). Evidence for systemic immune system alterations in sporadic amyotrophic lateral sclerosis (sALS). J Neuroimmunol.

[19] Turner MR, Goldacre R, Ramagopalan S, Talbot K, Goldacre MJ (2013). Autoimmune disease preceding amyotrophic lateral sclerosis: an epidemiologic study. Neurology..

[20] Lee DC, Rizer J, Selenica MLB, et al. LPS- induced inflammation exacerbates phospho-tau pathology in rTg4510 mice. *J Neuroinflammation*. 2010;7. 10.1186/1742-2094-7-56. 10.1186/1742-2094-7-56 PMC2949628 20846376

[21] Lee J, Lee Y, Yuk D (2008). Neuro-inflammation induced by lipopolysaccharide causes cognitive impairment through enhancement of beta-amyloid generation. J Neuroinflammation.

[22] Correia AS, Patel P, Dutta K, Julien JP. Inflammation induces TDP-43 mislocalization and aggregation. *PLoS One*. 2015;10(10). doi:10.1371/journal.pone.0140248. 10.1371/journal.pone.0140248 PMC4596857 26444430

[23] Fontaine SN, Zheng D, Sabbagh JJ (2016). DnaJ/Hsc70 chaperone complexes control the extracellular release of neurodegenerative-associated proteins. EMBO J.

[24] Iguchi Y, Eid L, Parent M (2016). Exosome secretion is a key pathway for clearance of pathological TDP-43. Brain..

[25] Carty N, Lee D, Dickey C (2010). Convection-enhanced delivery and systemic mannitol increase gene product distribution of AAV vectors 5, 8, and 9 and increase gene product in the adult mouse brain. J Neurosci Methods.

[26] Kitazawa M, Oddo S, Yamasaki TR, Green KN, LaFerla FM (2005). Lipopolysaccharide-induced inflammation exacerbates tau pathology by a cyclin-dependent kinase 5-mediated pathway in a transgenic model of Alzheimer’s disease. J Neurosci.

[27] Yasvoina MV, Genç B, Jara JH (2013). eGFP expression under UCHL1 promoter genetically labels corticospinal motor neurons and a subpopulation of degeneration-resistant spinal motor neurons in an ALS mouse model. J Neurosci.

[28] Swarup V, Phaneuf D, Dupré N (2011). Deregulation of TDP-43 in amyotrophic lateral sclerosis triggers nuclear factor κB-mediated pathogenic pathways. J Exp Med.

[29] Mackenzie IRA, Bigio EH, Ince PG (2007). Pathological TDP-43 distinguishes sporadic amyotrophic lateral sclerosis from amyotrophic lateral sclerosis with SOD1 mutations. Ann Neurol.

[30] Lu YC, Yeh WC, Ohashi PS (2008). LPS/TLR4 signal transduction pathway. Cytokine..

[31] Ayala YM, De Conti L, Avendaño-Vázquez SE (2011). TDP-43 regulates its mRNA levels through a negative feedback loop. EMBO J.

[32] Li W, Reeb AN, Lin B (2017). Heat shock-induced phosphorylation of TAR DNA-binding protein 43 (TDP-43) by MAPK/ERK kinase regulates TDP-43 function. J Biol Chem.

[33] Wils H, Kleinberger G, Janssens J (2010). TDP-43 transgenic mice develop spastic paralysis and neuronal inclusions characteristic of ALS and frontotemporal lobar degeneration. Proc Natl Acad Sci U S A.

[34] Tsai KJ, Yang CH, Fang YH (2010). Elevated expression of TDP-43 in the forebrain of mice is sufficient to cause neurological and pathological phenotypes mimicking FTLD-U. J Exp Med.

[35] Andrade-Moraes CH, Oliveira-Pinto AV, Castro-Fonseca E (2013). Cell number changes in Alzheimer’s disease relate to dementia, not to plaques and tangles. Brain..

[36] Blennow K, Bogdanovic N, Alafuzoff I, Ekman R, Davidsson P (1996). Synaptic pathology in Alzheimer’s disease: relation to severity of dementia, but not to senile plaques, neurofibrillary tangles, or the ApoE4 allele. J Neural Transm.

[37] Woo JAA, Liu T, Trotter C (2017). Loss of function CHCHD10 mutations in cytoplasmic TDP-43 accumulation and synaptic integrity. Nat Commun.

[38] Jiang T, Handley E, Brizuela M, et al. Amyotrophic lateral sclerosis mutant TDP-43 may cause synaptic dysfunction through altered dendritic spine function. *DMM Dis Model Mech*. 2019;12(5). doi:10.1242/dmm.038109. 10.1242/dmm.038109 PMC6550035 31036551

[39] Paolicelli RC, Jawaid A, Henstridge CM (2017). TDP-43 depletion in microglia promotes amyloid clearance but also induces synapse loss. Neuron.

[40] Badshah H, Ali T, Kim MO (2016). Osmotin attenuates LPS-induced neuroinflammation and memory impairments via the TLR4/NFκB signaling pathway. Sci Rep.

[41] Sheppard O, Coleman MP, Durrant CS (2019). Lipopolysaccharide-induced neuroinflammation induces presynaptic disruption through a direct action on brain tissue involving microglia-derived interleukin 1 beta. J Neuroinflammation.

[42] Xin Y-R, Jiang J-X, Hu Y (2019). The immune system drives synapse loss during lipopolysaccharide-induced learning and memory impairment in mice. Front Aging Neurosci.

[43] Jahn R, Südhof TC (1999). Membrane fusion and exocytosis. Annu Rev Biochem.

[44] Thammisetty SS, Pedragosa J, Weng YC, Calon F, Planas A, Kriz J (2018). Age-related deregulation of TDP-43 after stroke enhances NF-κB-mediated inflammation and neuronal damage. J Neuroinflammation.

[45] Cosenza-Nashat MA, Kim MO, Zhao ML, Suh HS, Lee SC (2006). CD45 isoform expression in microglia and inflammatory cells in HIV-1 encephalitis. Brain Pathol.

[46] Perlmutter LS, Scott SA, Barrón E, Chui HC (1992). MHC class II-positive microglia in human brain: Association with alzheimer lesions. J Neurosci Res.

[47] Nakano A, Harada T, Morikawa S, Kato Y (1990). Expression of leukocyte common antigen (CD45) on various human leukemia/lymphoma cell lines. Pathol Int.

[48] Holling TM, Schooten E, Van Den Elsen PJ (2004). Function and regulation of MHC class II molecules in T-lymphocytes: Of mice and men. Hum Immunol.

[49] Oh J, Shin JS (2015). Molecular mechanism and cellular function of MHCII ubiquitination. Immunol Rev.

[50] Joly-Amado A, Hunter J, Quadri Z (2020). CCL2 overexpression in the brain promotes glial activation and accelerates tau pathology in a mouse model of tauopathy. Front Immunol.

[51] Johnson EA, Dao TL, Guignet MA, Geddes CE, Koemeter-Cox AI, Kan RK. Increased expression of the chemokines CXCL1 and MIP-1α by resident brain cells precedes neutrophil infiltration in the brain following prolonged soman-induced status epilepticus in rats. *J Neuroinflammation*. 2011;8. 10.1186/1742-2094-8-41. 10.1186/1742-2094-8-41 PMC3104356 21535896

[52] Lee PY, Wang J-X, Parisini E, Dascher CC, Nigrovic PA (2013). Ly6 family proteins in neutrophil biology. J Leukoc Biol.

[53] Tsuiji H, Inoue I, Takeuchi M, et al. TDP-43 accelerates age-dependent degeneration of interneurons. *Sci Rep*. 2017;7(1). doi:10.1038/s41598-017-14966-w. 10.1038/s41598-017-14966-w PMC5668320 29097807

[54] Clark RE, Broadbent NJ, Squire LR (2005). Hippocampus and remote spatial memory in rats. Hippocampus..

[55] Cho YH, Kesner RP (1996). Involvement of entorhinal cortex or parietal cortex in long-term spatial discrimination memory in rats: Retrograde amnesia. Behav Neurosci.

[56] Cho YH, Beracochea D, Jaffard R (1993). Extended temporal gradient for the retrograde and anterograde amnesia produced by ibotenate entorhinal cortex lesions in mice. J Neurosci.

[57] Ramos JMJ (1998). Short communication: Retrograde amnesia for spatial information: a dissociation between intra and extramaze cues following hippocampus lesions in rats. Eur J Neurosci.

[58] Maviel T, Durkin TP, Menzaghi F, Bontempi B. Sites of neocortical reorganization critical for remote spatial memory. *Science (80- )*. 2004;305(5680):96-99. doi:10.1126/science.1098180. 10.1126/science.1098180 15232109

[59] Broadbent NJ, Squire LR, Clark RE (2006). Reversible hippocampal lesions disrupt water maze performance during both recent and remote memory tests. Learn Mem.

[60] Hok V, Save E, Lenck-Santini PP, Poucet B (2005). Coding for spatial goals in the prelimbic/infralimbic area of the rat frontal cortex. Proc Natl Acad Sci U S A.

[61] Basu J, Siegelbaum SA (2015). The corticohippocampal circuit, synaptic plasticity, and memory. Cold Spring Harb Perspect Biol.

[62] Genzel L, Battaglia FP. Cortico-hippocampal circuits for memory consolidation: the role of the prefrontal cortex. In: Springer, Cham; 2017:265-281. doi:10.1007/978-3-319-45066-7_16.

[63] De Bruin JPC, Swinkels WAM, De Brabander JM (1997). Response learning of rats in a Morris water maze: involvement of the medial prefrontal cortex. Behav Brain Res.

[64] De Bruin JPC, Moita MP, De Brabander HM, Joosten RNJMA (2001). Place and response learning of rats in a Morris water maze: differential effects of fimbria fornix and medial prefrontal cortex lesions. Neurobiol Learn Mem.

[65] Kolb B, Sutherland RJ, Whishaw IQ (1983). A comparison of the contributions of the frontal and parietal association cortex to spatial localization in rats. Behav Neurosci.

[66] Sang Jo Y, Eun HP, Il HK (2007). The medial prefrontal cortex is involved in spatial memory retrieval under partial-cue conditions. J Neurosci.

[67] Save E, Poucet B, Foreman N, Thinus-Blanc C (1998). The contribution of the associative parietal cortex and hippocampus to spatial processing in rodents. Psychobiology..

[68] Woolley DG, Laeremans A, Gantois I (2013). Homologous involvement of striatum and prefrontal cortex in rodent and human water maze learning. Proc Natl Acad Sci U S A.

[69] Nelson PT, Dickson DW, Trojanowski JQ (2019). Limbic-predominant age-related TDP-43 encephalopathy (LATE): consensus working group report. Brain..

[70] Hess DC, Bhutwala T, Sheppard JC, Zhao W, Smith J (1994). ICAM-1 expression on human brain microvascular endothelial cells. Neurosci Lett.

[71] Lutz SE, Smith JR, Kim DH (2017). Caveolin1 is required for Th1 cell infiltration, but not tight junction remodeling, at the blood-brain barrier in autoimmune neuroinflammation. Cell Rep.

[72] Haarmann A, Nowak E, Deiß A (2015). Soluble VCAM-1 impairs human brain endothelial barrier integrity via integrin α-4-transduced outside-in signalling. Acta Neuropathol.

[73] Blair LJ, Frauen HD, Zhang B (2015). Tau depletion prevents progressive blood-brain barrier damage in a mouse model of tauopathy. Acta Neuropathol Commun.

[74] Zlokovic BV (2008). The blood-brain barrier in health and chronic neurodegenerative disorders. Neuron..

[75] Daneman R (2012). The blood-brain barrier in health and disease. Ann Neurol.

[76] Banks WA, Robinson SM (2010). Minimal penetration of lipopolysaccharide across the murine blood-brain barrier. Brain Behav Immun.

[77] Liverani E, Rico MC, Yaratha L, Tsygankov AY, Kilpatrick LE, Kunapuli SP (2014). LPS-induced systemic inflammation is more severe in P2Y 12 null mice. J Leukoc Biol.

[78] Laurent C, Dorothée G, Hunot S, et al. Hippocampal T cell infiltration promotes neuroinflammation and cognitive decline in a mouse model of tauopathy. Brain. Published online 2017. doi:10.1093/brain/aww270. 10.1093/brain/aww270 PMC5382942 27818384

[79] Varvel NH, Neher JJ, Bosch A (2016). Infiltrating monocytes promote brain inflammation and exacerbate neuronal damage after status epilepticus. Proc Natl Acad Sci U S A.

[80] Stranahan AM, Hao S, Dey A, Yu X, Baban B (2016). Blood-brain barrier breakdown promotes macrophage infiltration and cognitive impairment in leptin receptor-deficient mice. J Cereb Blood Flow Metab.

[81] Crittenden M, Gough M, Harrington K, Olivier K, Thompson J, Vile RG (2003). Expression of inflammatory chemokines combined with local tumor destruction enhances tumor regression and long-term immunity. Cancer Res.

[82] Palmer C, Roberts RL, Young PI. Timing of neutrophil depletion influences long-term neuroprotection in neonatal rat hypoxic-ischemic brain injury. Pediatr Res. Published online 2004. doi:10.1203/01.PDR.0000113546.03897.FC. 10.1203/01.PDR.0000113546.03897.FC 14739365

[83] Werner C, Engelhard K. Pathophysiology of traumatic brain injury. Br J Anaesth. Published online 2007. doi:10.1093/bja/aem131. 10.1093/bja/aem131 17573392

[84] Zenaro E, Pietronigro E, Bianca V Della, et al. Neutrophils promote Alzheimer’s disease-like pathology and cognitive decline via LFA-1 integrin. *Nat Med*. Published online 2015. doi:10.1038/nm.3913. 10.1038/nm.3913 26214837

[85] Zhu J, Cynader MS, Jia W (2015). TDP-43 inhibits NF-κB activity by blocking p65 nuclear translocation. Scavone C, ed. PLoS One.

[86] Gulino R, Forte S, Parenti R, Gulisano M (2015). TDP-43 as a modulator of synaptic plasticity in a mouse model of spinal motoneuron degeneration. CNS Neurol Disord Drug Targets.

[87] Chen HJ, Mitchell JC, Novoselov S (2016). The heat shock response plays an important role in TDP-43 clearance: evidence for dysfunction in amyotrophic lateral sclerosis. Brain..

[88] Jinwal UK, Abisambra JF, Zhang J (2012). Cdc37/Hsp90 protein complex disruption triggers an autophagic clearance cascade for TDP-43 protein. J Biol Chem.

[89] Donnenfeld H, Kascsak RJ, Bartfeld H (1984). Deposits of IgG and C3 in the spinal cord and motor cortex of ALS patients. J Neuroimmunol.

[90] Engelhardt JI, Siklós L, Kőműves L, Smith RG, Appel SH (1995). Antibodies to calcium channels from ALS patients passively transferred to mice selectively increase intracellular calcium and induce ultrastructural changes in motoneurons. Synapse..

[91] Janelidze S, Hertze J, Nägga K (2017). Increased blood-brain barrier permeability is associated with dementia and diabetes but not amyloid pathology or APOE genotype. Neurobiol Aging.

[92] Bowman GL, Kaye JA, Moore M, Waichunas D, Carlson NE, Quinn JF (2007). Blood-brain barrier impairment in Alzheimer disease: Stability and functional significance. Neurology..

