# Supplementary figures and images for: TDP-43 mediated blood-brain barrier permeability and leukocyte infiltration promote neurodegeneration in a low-grade systemic inflammation mouse model
Source: J Neuroinflammation. 2020 Sep 26;17:283. doi: 10.1186/s12974-020-01952-9 (PMC7519496; doi:10.1186/s12974-020-01952-9)

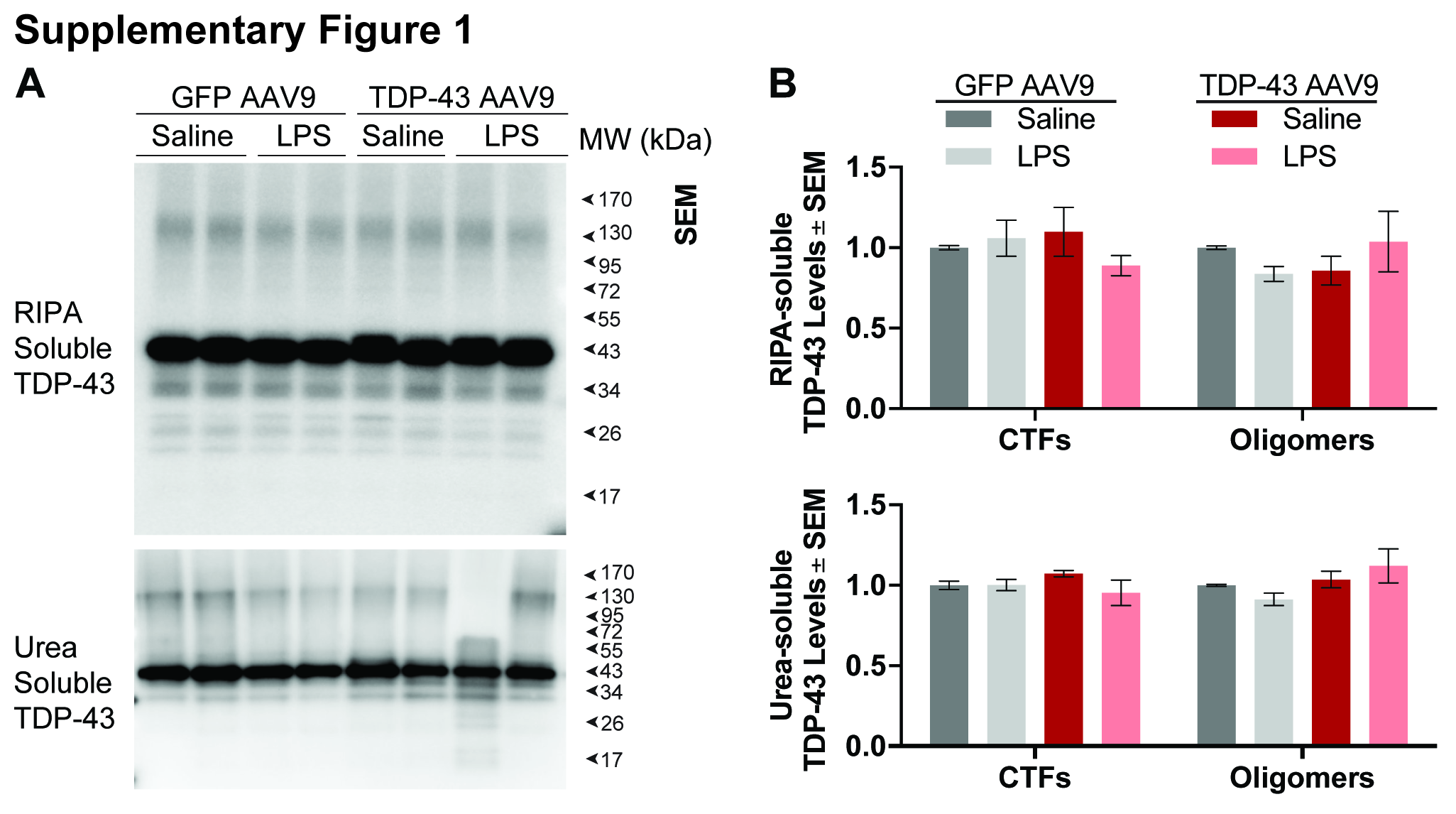

Supplement: Supplementary file 1 — Additional file 1: Figure S1. TDP-43 oligomers and c-terminal fragments are not altered by TDP-43 overexpression. (A) Western blots of RIPA and urea-soluble brain homogenates probed for total TDP-43 at a high exposure to visualize oligomers and C-terminal fragments. (B) Quantification of oligomers and C-terminal fragments in the RIPA and urea-soluble fractions (n = 4 mice/group). Statistical analysis was carried out using a one-way ANOVA with Bonferroni post-hoc test. [file 12974_2020_1952_MOESM1_ESM.tif]

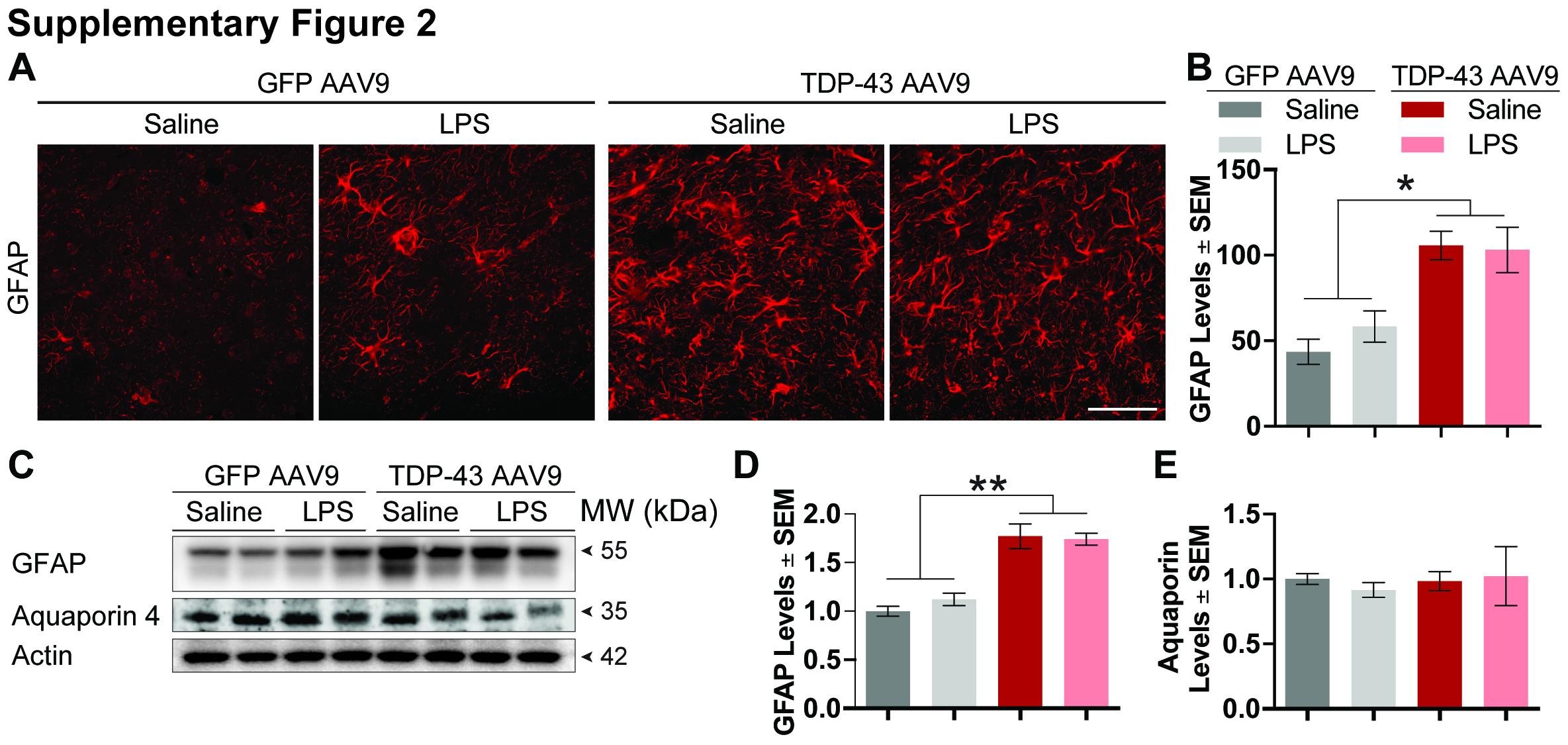

Supplement: Supplementary file 2 — Additional file 2: Figure S2. TDP-43 overexpression increases astrocytic activation. (A) Representative images depicting GFAP levels (red channel) in the frontal cortex of mouse brain tissue. Scale bar = 20 μm. (B) Quantification of GFAP levels. Statistical analysis was carried out using a one-way ANOVA with Bonferroni post-hoc test (n = 4 mice/group; * p < 0.05). (C) Representative blots probed for GFAP, aquaporin 4, and actin. (D) Quantification of GFAP and (E) aquaporin 4 levels normalized to actin. Statistical analysis was carried out using a one-way ANOVA with Bonferroni post-hoc test (n = 4 mice/group; ** p < 0.01). [file 12974_2020_1952_MOESM2_ESM.tif]

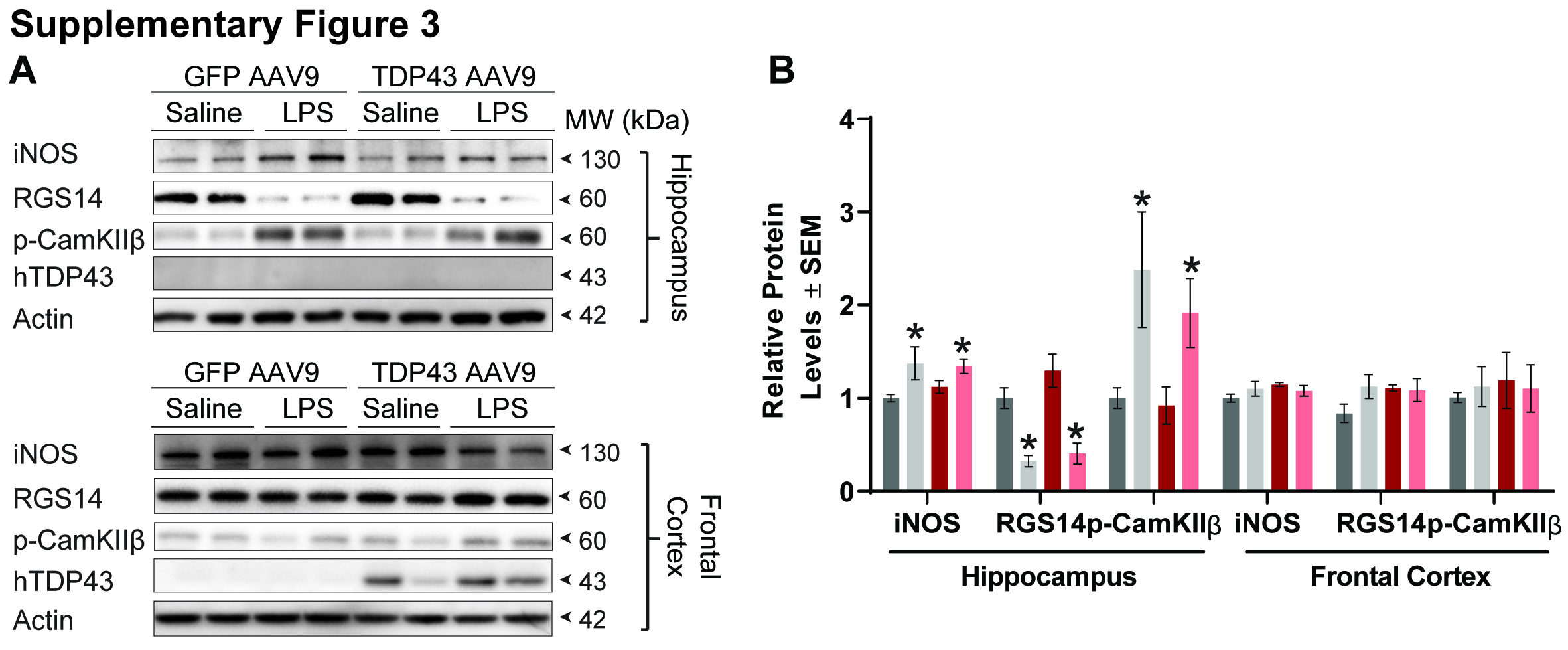

Supplement: Supplementary file 3 — Additional file 3: Figure S3. Regional differences in LPS-driven neuroinflammation. (A) Representative blots probed for p-CamKIIβ, RGS14, and iNOS in hippocampal and frontal cortex tissue lysates. (B) Quantification of p-CamKIIβ, RGS14, and iNOS normalized to actin. Statistical analysis was carried out using one-way ANOVAs with Bonferroni post-hoc test (n = 4 mice/group). [file 12974_2020_1952_MOESM3_ESM.tif]

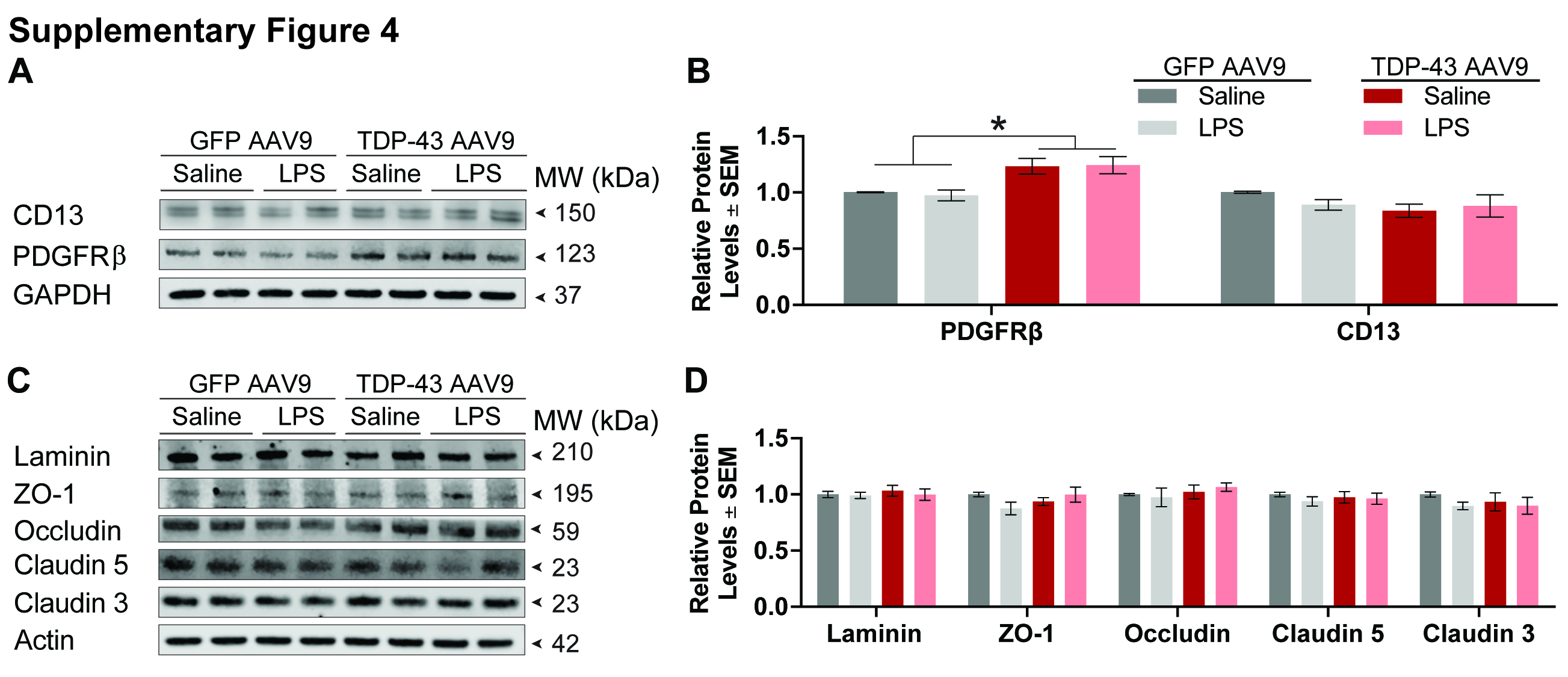

Supplement: Supplementary file 4 — Additional file 4: Figure S4. TDP-43 overexpression induces pericyte activation and does not alter tight junction protein levels. (A) Representative blots probed for CD13, PDGFRβ, and GAPDH. (B) Quantification of CD13 and PDGFRβ levels normalized to GAPDH. Statistical analysis was carried out using a one-way ANOVA with Bonferroni post-hoc test (n = 4 mice/group, * p < 0.05). (C) Representative blots probed for laminin B, ZO-1, occludin, claudin 5, claudin 3, and actin. (D) Quantification of laminin B, ZO-1, occludin, claudin 5, and claudin 3 levels normalized to actin. Statistical analysis was carried out using a one-way ANOVA with Bonferroni post-hoc test (n = 4 mice/group) [file 12974_2020_1952_MOESM4_ESM.tif]
